# Balanced crystalloids versus saline in the intensive care unit: study protocol for a cluster-randomized, multiple-crossover trial

**DOI:** 10.1186/s13063-017-1871-1

**Published:** 2017-03-16

**Authors:** Matthew W. Semler, Wesley H. Self, Li Wang, Daniel W. Byrne, Jonathan P. Wanderer, Jesse M. Ehrenfeld, Joanna L. Stollings, Avinash B. Kumar, Antonio Hernandez, Oscar D. Guillamondegui, Addison K. May, Edward D. Siew, Andrew D. Shaw, Gordon R. Bernard, Todd W. Rice, for the Isotonic Solutions and Major Adverse Renal Events Trial (SMART) Investigators, Gordon R. Bernard, Jonathan D. Casey, Matthew W. Semler, Michael J. Noto, Todd W. Rice, Daniel W. Byrne, Henry J. Domenico, Li Wang, Jesse M. Ehrenfeld, Jonathan P. Wanderer, Andrew D. Shaw, Antonio Hernandez, Avinash B. Kumar, Christopher G. Hughes, Emily Holcombe, Jayme Gibson, Lisa Weavind, Mias Pretorius, William T. Costello, Wesley H. Self, Edward D. Siew, Debra F. Dunlap, Joanna L. Stollings, Kelli A. Rumbaugh, Leanne Atchison, Mark Sullivan, Matthew Felbinger, Molly Knostman, Susan E. Hamblin, Addison K. May, Jason B. Young, Julie Y. Valenzuela, Oscar D. Guillamondegui, David P. Mulherin, Fred R. Hargrove, Seth Strawbridge, the Pragmatic Critical Care Research Group

**Affiliations:** 1 Division of Allergy, Pulmonary, and Critical Care Medicine, Vanderbilt University Medical Center, C-1216 MCN, 1161 21st Avenue South, Nashville, TN 37232-2650 USA; 2 Department of Emergency Medicine, Vanderbilt University Medical Center, Nashville, TN USA; 3 Department of Biostatistics, Vanderbilt University Medical Center, Nashville, TN USA; 4 Department of Anesthesiology, Vanderbilt University Medical Center, Nashville, TN USA; 5 Department of Biomedical Informatics, Vanderbilt University Medical Center, Nashville, TN USA; 6 Department of Surgery, Vanderbilt University Medical Center, Nashville, TN USA; 7 Department of Health Policy, Vanderbilt University Medical Center, Nashville, TN USA; 8 Department of Pharmaceutical Services, Vanderbilt University Medical Center, Nashville, TN USA; 9 Division of Nephrology and Hypertension, Vanderbilt Center for Kidney Disease (VCKD) and Vanderbilt Integrated Program for AKI Research (VIP-AKI), Vanderbilt University Medical Center, Nashville, TN USA

**Keywords:** Intravenous fluids, Crystalloid, Saline, Renal failure, Pragmatic trial

## Abstract

**Background:**

Saline, the intravenous fluid most commonly administered to critically ill adults, contains a high chloride content, which may be associated with acute kidney injury and death. Whether using balanced crystalloids rather than saline decreases the risk of acute kidney injury and death among critically ill adults remains unknown.

**Methods:**

The Isotonic Solutions and Major Adverse Renal Events Trial (SMART) is a pragmatic, cluster-level allocation, cluster-level crossover trial being conducted between 1 June 2015 and 30 April 2017 in five intensive care units at Vanderbilt University Medical Center in Nashville, TN, USA. SMART compares saline (0.9% sodium chloride) with balanced crystalloids (clinician’s choice of lactated Ringer’s solution or Plasma-Lyte A®). Each intensive care unit is assigned to provide either saline or balanced crystalloids each month, with the assigned crystalloid alternating monthly over the course of the trial. All adults admitted to participating intensive care units during the study period are enrolled and followed until hospital discharge or 30 days after enrollment. The anticipated enrollment is approximately 14,000 patients. The primary outcome is Major Adverse Kidney Events within 30 days—the composite of in-hospital death, receipt of new renal replacement therapy, or persistent renal dysfunction (discharge creatinine ≥200% of baseline creatinine). Secondary clinical outcomes include in-hospital mortality, intensive care unit-free days, ventilator-free days, vasopressor-free days, and renal replacement therapy-free days. Secondary renal outcomes include new renal replacement therapy receipt, persistent renal dysfunction, and incidence of stage 2 or higher acute kidney injury.

**Discussion:**

This ongoing pragmatic trial will provide the largest and most comprehensive comparison to date of clinical outcomes with saline versus balanced crystalloids among critically ill adults.

**Trial registration:**

For logistical reasons, SMART was prospectively registered separately for the medical ICU (SMART-MED; ClinicalTrials.gov identifier: NCT02444988; registered on 11 May 2015; date of first patient enrollment: 1 June 2015) and the nonmedical ICUs (SMART-SURG; ClinicalTrials.gov identifier: NCT02547779; registered on 9 September 2015; date of first patient enrollment: 1 October 2015).

**Electronic supplementary material:**

The online version of this article (doi:10.1186/s13063-017-1871-1) contains supplementary material, which is available to authorized users.

## Background

The administration of intravenous (IV) fluid is ubiquitous in the care of the critically ill [1]. Globally, 0.9% sodium chloride (saline) is the most common resuscitation fluid, but recent data have associated saline with hyperchloremia [2, 3], metabolic acidosis and renal vasoconstriction [4, 5], acute kidney injury (AKI) and renal replacement therapy (RRT) [6], and increased mortality [7, 8]. Although several observational studies [7, 9–11], a before-and-after trial [6], and meta-analyses [8, 12] suggested increased rates of AKI, RRT receipt, and death with saline compared with balanced crystalloids, researchers in two recent randomized pilot trials found no difference between crystalloids in any patient outcome [13, 14]. The number of patients enrolled in these pilot trials was insufficient to exclude small but potentially clinically meaningful differences in patient outcomes between saline and balanced crystalloids. Thus, the optimal choice of isotonic crystalloid for the treatment of critically ill adults remains unknown [15, 16]. To determine the impact of balanced crystalloids compared with saline on clinical outcomes among critically ill adults, a large, prospective, controlled trial is needed [13, 17].

The aim of the present trial is to compare the effect of balanced crystalloids with that of saline on the development of major adverse kidney events (the composite of death, new RRT, or persistent renal dysfunction) among intensive care unit (ICU) patients. Secondary aims are to evaluate the effect of balanced crystalloids with that of saline on laboratory values (serum chloride, serum bicarbonate, serum creatinine), organ injury (AKI, receipt of RRT), and additional clinical outcomes (ventilator-free days, ICU-free days, in-hospital mortality). We hypothesize that use of balanced crystalloids among ICU patients will reduce the incidence of major adverse kidney events.

## Methods

**Fig. 1:**
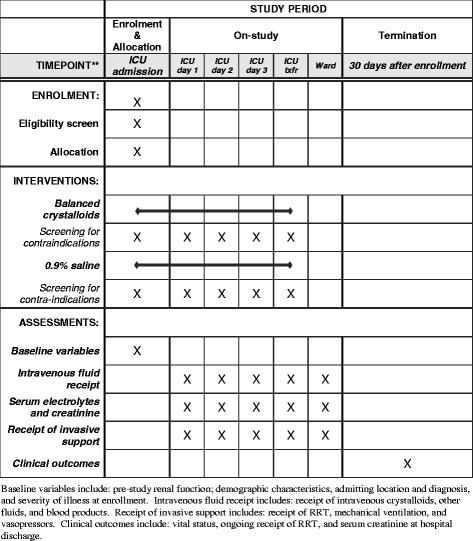
Standard Protocol Items: Recommendations for Interventional Trials (SPIRIT) checklist. Enrollment, interventions, and assessments. *ICU* Intensive care unit

This manuscript was written in accordance with Standard Protocol Items: Recommendations for Interventional Trials (SPIRIT) guidelines (see SPIRIT checklist in Additional file 1 and Fig. 1) [18].

**Fig. 2:**
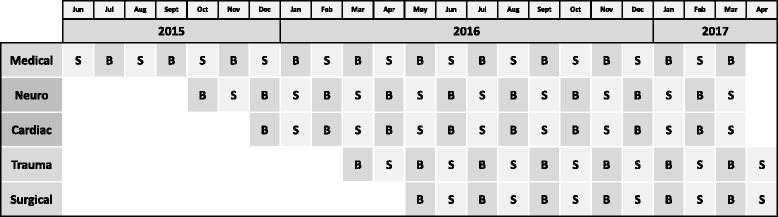
Crystalloid assignment during the trial. During each month of the study, each intensive care unit is assigned to use either 0.9% saline (S) or balanced crystalloids (B)

**Fig. 3:**
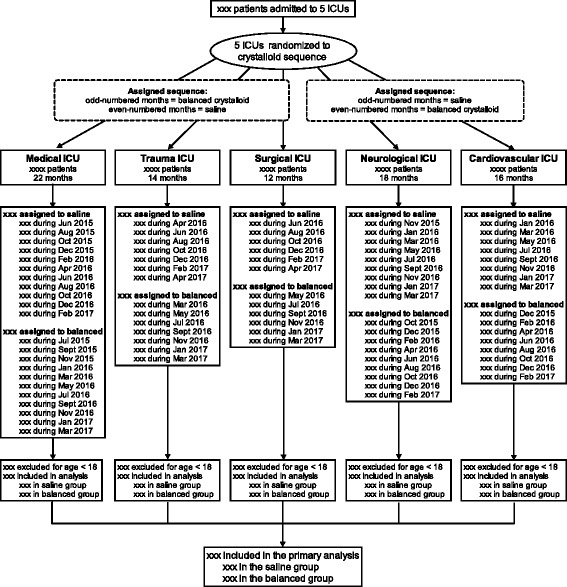
Flow diagram of progress of patients through the trial. *ICU* Intensive care unit

### Design

The Isotonic Solutions and Major Adverse Renal Events Trial (SMART) is a prospective, unblinded, pragmatic, cluster-level allocation, cluster-level crossover trial being conducted between 1 June 2015 and 30 April 2017 in five ICUs at Vanderbilt University Medical Center in Nashville, TN, USA. SMART compares saline (0.9% sodium chloride) with balanced crystalloids (lactated Ringer’s solution and Plasma-Lyte A® [Baxter Healthcare, Deerfield, IL, USA]) with regard to the primary outcome of Major Adverse Kidney Events within 30 days (MAKE30)—the composite of in-hospital death, receipt of new RRT, or persistent renal dysfunction (discharge creatinine ≥200% of baseline creatinine). Consistent with the concept of a pragmatic clinical trial [19, 20], the eligibility criteria are broad, the sample size is large, and study procedures are embedded into routine care and executed by clinical personnel. The trial was approved by the Vanderbilt University Medical Center Institutional Review Board (IRB) with waiver of informed consent (IRB 141349). The trial was registered with ClinicalTrials.gov prior to initiation of patient enrollment (ClinicalTrials.gov identifiers: NCT02444988, NCT02547779). An independent data and safety monitoring board (DSMB) is monitoring the progress and safety of the trial. The trial is investigator-initiated with funding provided by the Vanderbilt Institute for Clinical and Translational Research through a Clinical and Translational Science Award from the National Center for Advancing Translational Sciences (UL1 TR000445).

### Study sites and period

SMART is being conducted in five academic ICUs at Vanderbilt University Medical Center: a 34-bed medical ICU, a 22-bed neurological and neurosurgical ICU, a 27-bed cardiovascular ICU, a 31-bed trauma ICU, and a 22-bed surgical ICU. Participating ICUs began enrollment sequentially over the first year of the study (Fig. 2). Each ICU will enroll patients for at least 12 months and will enroll participants for an equal number of saline and balanced crystalloid months.

### Population

All adults (aged ≥18 years) admitted to a participating ICU at Vanderbilt University Medical Center during the study period are enrolled at the time of ICU admission. Enrolled patients who are discharged from the hospital are eligible again if they are admitted to a participating ICU again during the study period.

### Consent

Saline, lactated Ringer’s solution, and Plasma-Lyte A® are all IV crystalloids currently used in the routine care of patients admitted to the ICUs at Vanderbilt University Medical Center. Currently, no high-quality data suggest that choice of crystalloid affects clinical outcomes among critically ill adults. During the SMART trial, each time a study crystalloid is ordered, the study confirms that the treating clinician does not feel that a specific study crystalloid is required for the safe treatment of that specific patient at that specific point in time (see Study interventions section below). The trial is felt to pose minimal risk because (1) exposure to the study crystalloids occurs only for patients whose treating clinician has already decided to administer an IV crystalloid, (2) all of the crystalloid solutions examined are already used in routine practice in the study environment, (3) no definitive prior data suggest clinical outcomes are better with one crystalloid relative to the others, and (4) the study confirms with every crystalloid order that the treating clinician does not feel any one crystalloid type is required for safe treatment of the patient. Given the minimal risk, the focus of the study on crystalloid use at an ICU level, as well as the impracticability of consenting each patient admitted to each ICU prior to the first administration of crystalloid, a waiver of informed consent was granted by the Vanderbilt University Medical Center IRB (IRB 141349).

### Randomization and allocation

During each month of the study, each ICU is assigned to either saline or balanced crystalloids. So that each ICU would experience an equal number of months assigned to saline and balanced crystalloids while minimizing monthly imbalances in the hospital’s overall use of each crystalloid, we generated two sequences of study group assignment: (1) saline during odd-numbered months and balanced crystalloid during even-numbered months or (2) balanced crystalloid during odd-numbered months and saline during even-numbered months. We planned for three ICUs to be assigned to one sequence and the remaining two ICUs to the opposite sequence. To facilitate the early administration of the assigned crystalloid in the ED and operating room prior to the patient’s physical arrival in the ICU, a single, computer-generated, simple randomization was performed in which the three ICUs that admit the majority of patients from the ED (medical ICU, trauma ICU, and surgical ICU) were randomized en bloc to one sequence of crystalloid group assignments, and the two ICUs that admit the majority of patients from the operating room (neurological ICU and cardiac ICU) were randomized en bloc to the opposite sequence of crystalloid group assignments (Figs. 2 and 3).

### Concealment and blinding

Because available laboratory values overtly reflect the crystalloid being used, and because prior studies have shown high levels of provider awareness of crystalloid assignment despite attempts at blinding [13], patients, clinicians, and investigators are not blinded to crystalloid assignment. All study data, including the objective primary outcome, will be electronically extracted from the medical record in an automated manner unaffected by study group assignment.

### Study interventions

Study protocol determines only the choice of IV isotonic crystalloid: 0.9% sodium chloride (saline group) versus the treating clinician’s preference of lactated Ringer’s solution or Plasma-Lyte A® (balanced crystalloid group). The composition of each crystalloid solution is displayed in Additional file 2: Table S1. Lactated Ringer’s solution and Plasma-Lyte A® are the balanced crystalloids commonly available in the United States [21]. Lactated Ringer’s solution and Plasma-Lyte A® both offer a significantly lower chloride content than saline, but other minor differences in composition lead some clinicians to prefer one balanced crystalloid or the other for particular patients; for example, some clinicians prefer Plasma-Lyte A® over lactated Ringer’s solution for patients receiving blood transfusions [22]. Allowing clinicians to select either lactated Ringer’s solution or Plasma-Lyte A® when a balanced crystalloid is assigned is anticipated to improve compliance with balanced crystalloid assignment and emulate how balanced crystalloids are used in practice while maintaining relevant comparator groups consisting of crystalloid with a higher chloride content (saline) versus crystalloids with a lower chloride content (lactated Ringer’s solution and Plasma-Lyte A®). Decisions regarding crystalloid rate, volume, and additive content are deferred to treating clinicians.

Delivery of the assigned crystalloid to patients occurs via interventions in pharmacy supply and clinician order entry. Each month, the dispensing cabinets within the ICUs are stocked with 1000-ml bags of the assigned crystalloid. Additionally, any order for IV crystalloid for a patient located in a study ICU triggers an advisor application within the electronic order entry system. The advisor application informs providers about the study, asks about relative contraindications to the assigned crystalloid, and (if relative contraindications are not present) guides providers to order the assigned crystalloid. Accepted relative contraindications for patients assigned to balanced crystalloid include hyperkalemia and brain injury. The severity of hyperkalemia and brain injury at which saline will be used in favor of balanced crystalloids is determined by the treating clinician. The nonassigned crystalloid is also made available via the pharmacy if a formal statement is submitted that the attending physician feels the nonassigned crystalloid is required for the safe treatment of a specific patient.

Although the study is focused on crystalloid use in the ICU, crystalloid administration prior to ICU admission in the emergency department (ED) or operating room may introduce contamination and limit separation between study arms. Therefore, between 1 January 2016 and 30 April 2017, the Vanderbilt University Medical Center ED is coordinating their crystalloid use with the medical, surgical, and trauma ICUs such that patients admitted to those units from the ED begin receiving the assigned crystalloid during evaluation and management in the ED (ClinicalTrials.gov identifier: NCT02614040). Clinical outcomes of patients treated with study crystalloids in the ED and hospitalized outside the ICU will be recorded and reported separately. Similarly, to the extent that it is logistically feasible, for patients identified in the operating room as coming from or being admitted to one of the participating ICUs, the request is made that they receive the fluid assigned to the corresponding ICU during their operative procedure. Fluid administered prior to enrollment by the emergency medical system and outside hospitals, as well as fluid administered after discharge from the ICU, is not controlled by the study.

Each day patients receive the crystalloid to which their ICU is currently assigned. The necessity that an IV crystalloid be clinically available at all times precluded the use of washout periods, and patients who remain in the ICU through a crossover (i.e., from one calendar month to another) may potentially be exposed to both types of crystalloid. Although this introduces the potential for contamination of study groups, in a pilot trial at the same institution, the total volume of nonassigned crystalloid administered because of the lack of a washout period was <125 ml per patient [14]. As described in the Statistical analysis section below, patients will be analyzed in the group to which they were assigned at the time of study enrollment in an intention-to-treat fashion. For example, a patient admitted to an ICU during a month assigned to saline will be analyzed in the saline group even if that patient remains in the ICU after the ICU switches assignment to balanced crystalloids.

### Data collection

In this pragmatic trial, we are using data collected in routine clinical care and electronically extracted from the electronic health record (EHR) (see Additional file 2). All data are stored confidentially in an institutional patient data management system. Data collected include prestudy renal function; demographic characteristics, admitting location and diagnosis, and severity of illness at enrollment; receipt of IV crystalloids, other fluids, and blood products; serum electrolyte and creatinine values; receipt of RRT, mechanical ventilation, and vasopressors; and vital status and serum creatinine at hospital discharge. Electronic extraction of these data from the EHR has previously been validated against the reference standard of two-physician manual chart review [23]. For all patients who receive new RRT, study personnel will perform manual chart review to confirm the absence of prior RRT and identify the indication for RRT.

### Primary outcome

The primary outcome will be the proportion of patients meeting one or more criteria for MAKE30: in-hospital mortality, receipt of new RRT, or persistent renal dysfunction defined as a final inpatient serum creatinine value ≥200% of baseline [23–25]. In-hospital mortality will be defined as death due to any cause prior to hospital discharge censored at 30 days after ICU admission. Receipt of new RRT will be defined as receipt of any modality of RRT between ICU admission and the first of hospital discharge or 30 days among patients not known to have received RRT prior to ICU admission. Persistent renal dysfunction will be defined as a final serum creatinine value before hospital discharge (censored at 30 days after enrollment) ≥200% of the baseline creatinine value. The value for baseline serum creatinine will be determined using a previously described hierarchical approach [23]. The lowest serum creatinine between 12 months and 24 h prior to hospital admission will be used when available. If no such creatinine value is available, the lowest creatinine value between 24 h prior to hospital admission and the time of ICU admission will be used. If no creatinine value is available between 12 months prior to hospital admission and the time of ICU admission, a baseline creatinine value will be estimated using a previously described formula [creatinine = 0.74 − 0.2 (if female) + 0.08 (if African American) + 0.003 × age (in years)] [26]. Patients known to have received RRT prior to enrollment will be considered ineligible to meet criteria for new RRT or persistent renal dysfunction, but they may qualify for MAKE30 by experiencing in-hospital mortality.

### Secondary outcomes

Secondary outcomes will include additional clinical outcomes, additional renal outcomes, and biochemical outcomes. Additional clinical outcomes will include in-hospital mortality before ICU discharge, before 30 days, and before 60 days, as well as ICU-free days, ventilator-free days, vasopressor-free days, and RRT-free days, all through 28 days after enrollment. Additional renal outcomes will include new RRT receipt, persistent renal dysfunction, stage 2 or higher AKI according to Kidney Disease: Improving Global Outcomes (KDIGO) creatinine criteria [27], highest serum creatinine value, change from baseline creatinine to highest creatinine, final serum creatinine value before hospital discharge, and duration of new RRT. Biochemical outcomes will include serum values for sodium, potassium, chloride, bicarbonate, blood urea nitrogen, and creatinine from enrollment through day 30.

### Power calculation

On the basis of data from the study ICUs in the 1 year prior to the trial, we anticipate the planned study duration (Fig. 2) will result in enrollment of around 14,000 patients with an overall rate of MAKE30 around 15%. Enrollment of 14,000 patients will provide 90% power at an α level of 0.05 to detect an absolute difference between the saline and balanced crystalloid groups in MAKE30 of 1.9%, as well as a relative risk reduction of 12%, which is comparable to the 12% relative risk reduction for in-hospital mortality reported in a recent pilot trial [13] (additional details in Additional file 2).

### Data and safety monitoring board and interim analysis

A DSMB was appointed to oversee the conduct of the trial and review two interim analyses. The DSMB is comprised of two academic intensivists outside the study institution who are experienced in the conduct of clinical trials in critical illness. The first interim analysis occurred 6 months after study initiation, examining patients enrolled between 1 June 2015 and 30 November 2015. The second interim analysis occurred halfway between the first interim analysis and the end of the trial, examining patients enrolled between 1 June 2015 and 31 July 2016 (additional details in Additional file 2). Both interim analyses used the same stopping criteria:

*The stopping boundary for efficacy will be met if (1) the unadjusted difference in the incidence of the primary outcome (MAKE30) between study groups is greater than or equal to 2.6% with a* *P* *value less than 0.001 and (2) the* *P* *value is less than 0.001 for the difference between study groups in the incidence of either in-hospital mortality or receipt of new RRT. Because even small differences between groups would be clinically meaningful, and given the importance of determining with as much certainty as possible whether balanced crystalloids are superior to saline, a futility stopping boundary will not be employed. Use of the conservative Haybittle-Peto boundary* (*P* *< 0.001) will allow the final analysis to be performed using an unchanged level of significance* (*P* *= 0.05).*

At the time of submission of the manuscript of this report, both interim analyses had been completed, and the DSMB had recommended continuing the trial to completion. In addition, the DSMB is available to evaluate adverse events or serious adverse events during the conduct of the trial. In cases of serious adverse events, the DSMB has the ability to pause the trial to investigate possible safety issues and suggest changes to the design of the study to abrogate any safety issues.

### Statistical analysis principles

All analyses will be performed using R version 3.2.0 software (R Foundation for Statistical Computing, Vienna, Austria). To maximize transparency and reproducibility, a complete version of the R code that will be used to analyze the final study data is available in Additional file 3. This ensures that (1) statistical reviewers or external investigators will be able to replicate the prespecified analysis of the trial independently and (2) any changes or additions to the statistical analysis introduced by investigators or reviewers after completion of enrollment will be evident as differences between the prespecified code and the analysis code included with the final publication.

All analyses will be conducted at the level of the individual patient during an individual hospitalization in an intention-to-treat fashion unless otherwise specified. Continuous variables will be reported as mean ± SD, mean and 95% CI, or median and IQR; categorical variables will be reported as frequencies and proportions. Between-group comparisons will be made with the Mann-Whitney rank-sum test for continuous variables, the chi-square test for categorical variables, generalized estimating equations for repeatedly measured variables, and generalized linear mixed-effects models for analyses of the primary and secondary outcomes. A two-sided *P* value <0.05 will be considered statistically significant.

### Analytic rationale

In the setting of a large, pragmatic trial enrolling every adult admitted to the five participating ICUs, the SMART study population will contain a wide spectrum of (1) exposure to the study intervention, (2) baseline risk of the primary outcome, and (3) physiologically distinct patient subgroups. The primary and secondary analyses evaluate the effect of the intervention overall and across the spectrum of exposure to crystalloid, baseline risk of MAKE30, and patient subgroups.

### Primary analysis

To account for the cluster-level allocation, cluster-level crossover structure of the trial, the primary analysis will be an intention-to-treat comparison of the primary outcome of MAKE30 between the saline and balanced crystalloid groups using a generalized linear mixed-effects model including fixed effects (group assignment, age, sex, race, source of admission, mechanical ventilation, vasopressor receipt, diagnosis of sepsis, and diagnosis of traumatic brain injury) and random effects (ICU) (additional details in Additional file 2) [28, 29].

### Main secondary analysis

Anticipating (1) a wide range in the total volume of crystalloid received by study participants and (2) the potential for greater difference in outcomes between study groups among those patients who receive larger volumes of crystalloid, the main secondary analysis will compare the proportion of patients experiencing MAKE30 in the saline and balanced crystalloid groups, accounting for patients’ overall volume of isotonic crystalloid received. For this analysis, we will construct a logistic regression model with MAKE30 as the outcome and independent variables of study group, total isotonic crystalloid received between enrollment and 30 days, and the interaction between the two (as a cross-product term). This will allow us to determine whether any volume of crystalloid receipt exists at which use of balanced crystalloids decreases the risk of MAKE30 compared with saline.

Given that total crystalloid receipt is a variable that emerges after enrollment, we will perform sensitivity analyses (1) using total crystalloid receipt in the 72 h after enrollment (before incident AKI or death are likely to have affected isotonic crystalloid administration), (2) replacing the actual total crystalloid receipt with predicted total crystalloid receipt based on a multivariable linear regression model using patient and ICU characteristics available at the time of enrollment derived from crystalloid administration in the study ICUs in the 1 year prior to the trial, and (3) comparing outcomes between study groups among a modified intention-to-treat population of patients who received at least 500 ml of any study crystalloid in the 72 h after enrollment.

### Additional secondary analyses

We will perform the following additional secondary analyses:

- *Comparison of secondary outcomes between study groups.*
- *Effect modification by severity of illness and prespecified subgroups.* Using generalized linear mixed-effects modeling, we will examine the interaction between crystalloid assignment and the following baseline variables with respect to the primary outcome of MAKE30 in the intention-to-treat population:
- Source of admission to the ICU (ED, operating room, transfer from another hospital, hospital ward, other)
- Study ICU (medical, surgical, cardiac, neurological, trauma) (Because cluster cannot be treated as a random effect for this subgroup, we will use logistic regression modeling.)
- Sepsis or septic shock (yes, no)
- Traumatic brain injury (yes, no)
- Receipt of mechanical ventilation (yes, no)
- Receipt of vasopressors (yes, no)
- Category of renal dysfunction at the time of enrollment (no renal dysfunction, AKI, chronic kidney disease, end-stage renal disease receiving RRT)
- Risk of in-hospital mortality as predicted by baseline University HealthSystem Consortium expected in-hospital mortality (continuous variable ranging from 0.0 to 1.0)
- *Sensitivity analysis excluding patients admitted in the week prior to a crossover (“washout”).* We will repeat the primary analysis comparing MAKE30 between study groups in the intention-to-treat population excluding those admitted in the 7 days prior to a crossover in ICU crystalloid assignment (simulating a washout period). Prior data from the study ICUs suggest that less than 10% of patients remain in the ICU for longer than 7 days [14]. Excluding those admitted within 7 days of a crossover in ICU crystalloid assignment will allow use of a baseline factor to exclude the majority of patients who would go on to experience a crossover in crystalloid assignment because of the study design.
- *Sensitivity analysis excluding patients who were transferred between ICUs or remained in the ICU through a crossover (“per protocol”).* We will repeat the primary analysis comparing MAKE30 between study groups in the intention-to-treat population excluding those who remained in the ICU through a crossover in crystalloid assignment or who were transferred between study ICUs.
- *Sensitivity analysis including only each patient’s first admission to a participating ICU during the study period.* We will repeat the primary analysis comparing MAKE30 between study groups in the intention-to-treat population including only the first ICU admission in the study for each patient.

### Corrections for multiple testing

All of the additional secondary analyses will be considered hypothesis-generating, and no corrections for multiple comparisons will be performed.

### Handling of missing data

Of the components of the MAKE30 primary outcome, data regarding in-hospital mortality and receipt of new RRT are not anticipated to be missing for any patients [14, 23]. In contrast, the persistent renal dysfunction component of MAKE30 may suffer from missing data for serum creatinine value at baseline or between enrollment and hospital discharge. In a pilot study of 974 patients in the same hospital, 31 patients (3.2%) had no measured serum creatinine between enrollment and hospital discharge [14]. Of these 31 patients, 6 (19.4%) died within hours of ICU admission and qualified for the MAKE30 outcome via the in-hospital mortality criteria. The remaining 25 (80.6%) were low-acuity ICU patients with a normal creatinine value measured in the 24 h prior to ICU admission who were discharged from the hospital within 48 h without another serum creatinine measurement. Of these, 24 had a subsequent outpatient serum creatinine value measured in the next 90 days, all of which measurements were in the normal range. Thus, patients without a serum creatinine measurement between enrollment and hospital discharge who do not experience in-hospital mortality or new RRT will be classified as not having experienced the MAKE30 outcome.

With regard to missing data for baseline serum creatinine, in the same pilot study, 595 (61.0%) of 974 patients had a measured serum creatinine value between 12 months and 24 h prior to hospital admission [14]. Of those without such a measurement, 259 (68.3%) of 379 had a value measured between 24 h prior to hospital admission and study enrollment. Only 120 (12.3%) of 974 patients did not have an available serum creatinine value prior to enrollment. For the main analysis, patients without a measured serum creatinine value between 12 months prior to hospital admission and enrollment will have a baseline creatinine value estimated using a previously described three-variable formula [26]. Multiple alternative approaches to missing baseline creatinine data will be explored in sensitivity analyses, including use of complete cases, multivariable single imputation, and use of the first creatinine after enrollment or the highest or lowest creatinine during the study (see Additional file 2).

### Post hoc analyses

In the event that investigators or reviewers introduce analyses in addition to those described above, these will be clearly delimitated as post hoc and will be considered hypothesis-generating.

### Presentation of the results

After completion of enrollment and data analysis, the results of the trial will be communicated to the public through manuscript publication and submission of the results to the ClinicalTrials.gov database. Submission for publication will include public access to the full study protocol and statistical code. Authorship will be based on the International Committee of Medical Journal Editors guidelines, and professional writers will not be used.

The flow of patients through the study will be presented in a flow diagram (Fig. 3). Baseline characteristics will be presented by treatment group, as shown in Table 1 and Additional file 2: Table S2. The volume of isotonic crystalloid administered, other fluids, and blood products administered over time will be presented by treatment group (Additional file 2: Table S3). Serum values for sodium, potassium, chloride, bicarbonate, blood urea nitrogen, and creatinine will be presented in figures displaying serum values over time by group and in tables detailing the incidence of abnormal values (Additional file 2: Table S4). Clinical and renal outcomes will be reported by treatment group, as shown in Table 2. For the primary analysis of the primary outcome, we will present the unadjusted frequency and proportion of MAKE30 in each study group, as well as the adjusted OR, 95% CI, and *P* value derived from the generalized linear mixed-effects model. Indications for new RRT are displayed as in Additional file 2: Table S5. Heterogeneity of treatment effect analyses will be displayed as locally weighted scatterplot smoothing (LOESS) curves or partial effect plots for continuous variables and forest plots for categorical variables.

**Table 1:** Patient characteristics at baseline

Patient characteristics	Saline (*n* =)	Balanced (*n* =)
Age, years, median [IQR]	–	–
Male sex, *n* (%)	–	–
White race, *n* (%)	–	–
Weight, kg, median [IQR]	–	–
Body mass index, kg/m^2^, median [IQR]	–	–
Renal comorbidities, *n* (%)		
Chronic kidney disease, stage 3 or higher	–	–
Prior RRT receipt	–	–
Source of admission to ICU, *n* (%)		
Emergency department	–	–
Transfer from another hospital	–	–
Hospital ward	–	–
Another ICU within the hospital	–	–
Operating room	–	–
Outpatient	–	–
Study ICU, *n* (%)		
Medical	–	–
Surgical	–	–
Cardiac	–	–
Neuro	–	–
Trauma	–	–
Admitting diagnosis, *n* (%)	–	–
Sepsis or septic shock	–	–
Traumatic brain injury		
Mechanical ventilation, *n* (%)	–	–
Vasopressors, *n* (%)	–	–
UHC expected mortality, %, mean (95% CI)	–	–
Serum creatinine, mg/dl, median [IQR]		
Lowest in 12 months prior to hospitalization	–	–
No. (%) of patients	–	–
Lowest between hospitalization and ICU admission	–	–
No. (%) of patients	–	–
Estimated by three-variable formula	–	–
No. (%) of patients	–	–
Study baseline	–	–
Acute kidney injury, stage 2 or higher	–	–

*ICU* Intensive care unit, *UHC* University HealthSystem Consortium

**Table 2:** Clinical outcomes

Outcome	Saline (*n* =)	Balanced (*n* =)	Adjusted OR (95% CI)	Adjusted *P* value
Primary outcome				
Major Adverse Kidney Event within 30 days, *n* (%)	–	–	–	–
Secondary clinical outcomes				
In-hospital mortality, *n* (%)				
Before ICU discharge	–	–	–	–
Before 30 days	–	–	–	–
Before 60 days	–	–	–	–
ICU-free days, median [IQR]	–	–	–	–
Mean ± SD	–	–		
Ventilator-free days, median [IQR]	–	–	–	–
Mean ± SD	–	–		
Vasopressor-free days, median [IQR]	–	–	–	–
Mean ± SD	–	–		
RRT-free days, median [IQR]	–	–	–	–
Mean ± SD	–	–		
Secondary renal outcomes				
Serum creatinine, mg/dl				
Highest before discharge or day 30, mg/dl, median [IQR]	–	–	–	–
Change from baseline to highest value, mg/dl, median [IQR]	–	–	–	–
Final value before discharge or 30 days, mg/dl, median [IQR]	–	–	–	–
Among survivors, mg/dl, median [IQR]	–	–	–	–
Final creatinine ≥200% baseline, *n* (%)	–	–	–	–
Among survivors to hospital discharge	–	–	–	–
Among survivors to hospital discharge without new RRT	–	–	–	–
Acute kidney injury, stage 2 or higher, *n* (%)	–	–	–	–
Developing after enrollment	–	–	–	–
Receipt of new RRT, No. (%)	–	–	–	–
Duration of in-hospital receipt, days, median [IQR]	–	–	–	–
Continued receipt after hospital discharge, *n* (%)	–	–	–	–

*ICU* Intensive care unit, *RRT* Renal replacement therapy

## Discussion

Upon completion, SMART will provide the most comprehensive data to date on the comparative effects of saline versus balanced crystalloids among critically ill adults. Given that isotonic crystalloid administration represents the most common intervention provided to hospitalized patients, saline and balanced crystalloids are the only available options for isotonic crystalloid administration, and also that the relationship between saline and AKI and death remains unclear, the results of SMART will have immediate implications for the care of a broad population of acutely ill patients. Results showing superior clinical outcomes in the balanced crystalloids group would provide compelling evidence that balanced solutions should be considered the preferred isotonic crystalloid for most acutely ill patients. Better clinical outcomes with saline would cement 0.9% sodium chloride as the first-line isotonic IV fluid and end the current debate about optimal crystalloid composition. In this comparative effectiveness trial of thousands of critically ill adults, a finding of no difference between groups would still have important implications for clinical care and future research. In a trial powered to detect absolute risk reductions as small as 2% in clinical outcomes, no difference between groups would imply that the effect of crystalloid choice for the majority of ICU patients is minimal, and any future research would need to be focused on select subpopulations.

While designing SMART, we weighed the relative advantages and disadvantages of multiple study designs, including a blinded, patient-level randomized trial. A major challenge to controlled studies of fluid administration in critical illness is the ability to enroll patients prior to the period of highest fluid exposure. Because the majority of fluid is administered as part of resuscitation in the ED and during the first 12 h of ICU admission, we selected a cluster-level allocation design that would allow enrollment immediately upon presentation and coordination between study ICUs and the ED to maximize exposure to the assigned crystalloid and minimize exposure to the nonassigned crystalloid. By basing study group assignment at the unit level, we ensured delivery of the assigned crystalloid even among unstable patients for whom fluid was being administered immediately upon presentation, because the assigned crystalloid would be the fluid most readily available in the study unit. The enrollment of all adults admitted to the participating ICUs examines the effects of saline versus balanced crystalloids in a real-world clinical environment, improving the generalizability of the study findings. Coupling group assignment at the level of the ICU with relatively short periods (1 month) and frequent crossovers (at least 11 in each unit) balances baseline characteristics and cointerventions better than a simple cluster-randomized trial or before-and-after trial with the same number of units, decreasing confounding by seasonal change or trends in usual care over time. Although blinding of treating clinicians and study personnel to the assigned intervention would be ideal, researchers in a prior pilot trial of the same topic found high rates of provider awareness of crystalloid assignment despite blinding, perhaps owing to the overt effect of the study crystalloids on clinically available laboratory values such as serum chloride and bicarbonate [13]. Use of an objective, patient-centered primary outcome abstracted automatically from the EHR increases the pragmatic nature of the design and diminishes the risk of observer bias.

Several potential threats to the validity of our trial exist. Including all patients admitted to each study ICU may produce a patient population with limited average exposure to the study interventions [13, 14]. On the basis of our preliminary data from the same units prior to this study, however, we anticipate that more than 90% of enrolled patients will receive isotonic crystalloid and at least 25% of patients will receive more than 4 L of isotonic crystalloid, which is comparable to or greater than that received in prior positive ICU fluid trials [30]. Additionally, we have prespecified analyses to evaluate for a dose-response relationship between the volume of isotonic crystalloid administered and clinical outcomes with saline versus balanced crystalloid. Similarly, the broad enrollment criteria may produce a study population at relatively low risk for adverse clinical outcomes. The anticipated incidence of the primary outcome of 15%, however, is comparable to that of other large ICU fluid management trials [30, 31]. Treating clinicians are aware of study group assignment, which may permit a treatment bias in which clinicians administer less isotonic crystalloid and/or more nonisotonic crystalloids when assigned to one of the fluid groups. For this reason, we will record and report use of not only isotonic crystalloid but also nonisotonic crystalloid, colloid, and blood products during the trial. Group assignment at the level of the cluster with multiple cluster-level crossovers introduces the possibility for intracluster correlation, interperiod correlation, and intracluster intraperiod correlation, which may confound the relationship between group assignment and clinical outcome. In preparatory analyses using data from more than 10,000 patients admitted in the 1 year prior to the trial, we found the effect of intracluster correlation to be minimized by the short periods and frequent crossovers and the effects of intraperiod correlation and intracluster intraperiod correlation to be small (see Additional file 2: Supplemental methods). Our primary analysis uses a generalized linear mixed-effects model to account for these aspects of the study structure. In the absence of a washout period, there will be carryover of crystalloid administration from one group assignment into the other; however, on the basis of pilot data, we anticipate the volume of nonassigned crystalloid received as a result of carryover will be low [14], and we prespecify secondary analyses to address the effects of carryover. Finally, although MAKE30 is a recommended outcome for clinical trials involving AKI [24, 32], use of a composite outcome presents potential challenges. Unlike death and new receipt of RRT, whether persistent renal dysfunction on hospital discharge is a patient-centered outcome remains a point of discussion. Persistent renal dysfunction also relies on the availability of serum creatinine measurements at baseline and before hospital discharge, potentially requiring imputation of missing data for one component of the composite primary outcome. Perhaps most important, although death, new receipt of RRT, and persistent renal dysfunction are weighted equally in the MAKE30 composite outcome, they may not represent equivalent outcomes to patients or providers. To address this, we will provide data on the MAKE30 outcome overall and for each of its separate components.

## Trial status

SMART is an ongoing, pragmatic, cluster-level allocation, cluster-level crossover trial that will compare saline to balanced crystalloids with regard to major adverse kidney events among critically ill adults. Patient enrollment began on 1 June 2015, and enrollment is scheduled for completion on 30 April 2017.

## Additional files

Additional file 1: SPIRIT 2013 checklist: recommended items to address in a clinical trial protocol and related documents. (DOC 122 kb)
Additional file 2: This file contains supplemental tables and methods, including additional details regarding electronic health record-based data collection, power calculation, development of the model for the primary analysis, interim analyses, and handling of missing data for baseline creatinine. (DOCX 88 kb)
Additional file 3: This file contains a .pdf version of the R code that will be used to analyze the final study data. (PDF 118 kb)

## Abbreviations

AKI: Acute kidney injury
DSMB: Data and safety monitoring board
ED: Emergency department
EHR: Electronic health record
ICU: Intensive care unit
IRB: Institutional review board
IV: Intravenous
KDIGO: Kidney Disease: Improving Global Outcomes
LOESS: Locally weighted scatterplot smoothing
MAKE30: Major Adverse Kidney Events within 30 days
RRT: Renal replacement therapy
SMART: Isotonic Solutions and Major Adverse Renal Events Trial
SPIRIT: Standard Protocol Items: Recommendations for Interventional Trials
UHC: University HealthSystem Consortium
