# Supplementary material for: Balanced crystalloids versus saline in the intensive care unit: study protocol for a cluster-randomized, multiple-crossover trial
Source: Trials. 2017 Mar 16;18:129. doi: 10.1186/s13063-017-1871-1 (PMC5356286; doi:10.1186/s13063-017-1871-1)
Supplement: Additional file 2: — This file contains supplemental tables and methods, including additional details regarding electronic health record-based data collection, power calculation, development of the model for the primary analysis, interim analyses, and handling of missing data for baseline creatinine. (DOCX 88 kb) [file 13063_2017_1871_MOESM2_ESM.docx]

**Balanced Crystalloids versus Saline in the Intensive Care Unit: Study Protocol for a Cluster-Randomized, Multiple-Crossover Trial**

Matthew W. Semler, MD, MSc; Wesley H. Self, MD, MPH; Li Wang, MS; Daniel W. Byrne, MS; Jonathan P. Wanderer, MD, MPhil; Jesse M. Ehrenfeld, MD, MPH; Joanna L. Stollings, PharmD, FCCM, FCCP; Avinash B. Kumar, MD, FCCM, FCCP; Antonio Hernandez, MD, MSc; Oscar D. Guillamondegui, MD; Addison K. May, MD, FACS; Edward D. Siew, MD, MSc; Andrew D. Shaw, MB, FRCA; Gordon R. Bernard, MD; Todd W. Rice, MD, MSc for the SMART Investigators and the Pragmatic Critical Care Research Group

Online Data Supplement

**SUPPLEMENTAL METHODS**A. Definitions of Study Variables

B. Electronic Health Record-based Data Collection

C. Power Calculation

D. Development of the Model for the Primary Analysis

E. Interim Analyses

F. Handling of Missing Baseline Creatinine

**SUPPLEMENTAL TABLES**

Table S1. Composition of the study fluids.

Table S2. Elixhauser comorbidity index.

Table S3. Intravenous fluids and blood products.

Table S4. Serum laboratory values.

Table S5. Indications for new renal replacement therapy.

**SUPPLEMENTAL REFERENCES**

**SUPPLEMENTAL METHODS**

**A. Definitions of Study Variables**

***Fluids***

**Intravenous fluid** – For the SMART study, intravenous fluid will be defined as the intravenous administration of any formulation of any volume at any rate of 0.9% sodium chloride, Lactated Ringer’s, Plasma-Lyte A®; 0.45% sodium chloride, 0.225% sodium chloride, dextrose in water, 20% or 5% human albumin solution, gelatins, dextrans, or hydroxyethyl starches. This will include fluid given as a bolus, fluid given as maintenance infusions, fluid given as flushes, fluid given as ‘piggy-back’ infusions for IV medications, fluid given through pressure-bag systems, fluid given as a part of thermodilution of pulmonary artery catheters, and fluid given to maintain the patency of peripheral venous access. This will not include carrier fluid for medications and oral fluids.

**Isotonic crystalloid** – For the SMART study, the term isotonic crystalloid will be used to refer to any of 0.9% sodium chloride, Lactated Ringer’s, or Plasma-Lyte A®. Use of the term isotonic crystalloid is intended to distinguish these three fluids from colloid solutions and from significantly hypotonic (0.45% sodium chloride) or hypertonic (3% sodium chloride) crystalloid solutions, rather than to imply that the tonicities of 0.9% sodium chloride, Lactated Ringer’s, or Plasma-Lyte A® are precisely comparable to extracellular fluid.

**Saline** – Saline will refer to 0.9% sodium chloride.

**Balanced Crystalloid** – For the SMART study, Lactated Ringer’s or Plasma-Lyte A® will be referred to as balanced crystalloids.

***Renal Function***

**Baseline serum creatinine** – The value for baseline serum creatinine will be determined in a hierarchical approach. The lowest serum creatinine between 12 months and 24 h prior to hospital admission will be used when available. If no such creatinine value is available, the lowest creatinine value between 24 h prior to hospital admission and the time of ICU admission will be used. If no creatinine value is available between 12 months prior to hospital admission and the time of ICU admission, a baseline creatinine value will be estimated using a previously-described three-variable formula [creatinine = 0.74 − 0.2 (if female) + 0.08 (if African American) + 0.003 × age (in years)]^1^.

**Acute kidney injury, stage II or greater** – Stage II or greater acute kidney injury will be defined according to Kidney Disease Improving Global Outcomes (KDIGO) creatinine criteria^2^ as a creatinine value between enrollment and the first of hospital discharge or 30 days at least 200% of the baseline value OR both (1) greater than 4.0 mg/dL and (2) increased at least 0.3 mg/dL from baseline. Patients may have acute kidney injury present at the time of first creatinine measurement after enrollment (prevalent AKI) or acute kidney injury developing during the study (incident AKI). Incident AKI will be defined as any creatinine value between enrollment and discharge or 30 days that is (1) increased at least 0.3 mg/dL from a preceding post-enrollment value AND (2) at least 200% of the baseline value, at least 200% of a preceding post-enrollment value, or at least 4.0 mg/dL.

**Chronic kidney disease stage III or greater** – Chronic kidney disease stage III or greater will be defined as a glomerular filtration rate less than 60 ml/min per 1.73 m^2^ as calculated by the Chronic Kidney Disease Epidemiology (CKD-EPI) Collaboration equation^3^ using the patient’s baseline creatinine value.

***Outcomes***

**Major Adverse Kidney Events within 30 days (MAKE30).** The MAKE30 composite outcome will be considered to have occurred when patients meet one or more of the following criteria in the 30 days after enrollment: (1) in-hospital mortality, (2) receipt of new renal replacement therapy (RRT), or (3) persistent renal dysfunction. Patients who have received RRT prior to enrollment will be ineligible to meet the new RRT or persistent renal dysfunction criteria but will remain eligible to meet criteria for in-hospital mortality.

**In-hospital mortality** – In-hospital mortality will be defined as death from any cause prior to hospital discharge censored at 30 days after ICU admission (30-day in-hospital mortality).

**Receipt of new renal replacement therapy** – Receipt of new RRT will be defined as receipt of any modality of RRT between ICU admission and the first of (1) hospital discharge or (2) 30 days in a patient not known to have received RRT prior to ICU admission.

**Persistent renal dysfunction** – Persistent renal dysfunction will be defined as a final serum creatinine value before hospital discharge (censored at 30 days after enrollment) that is ≥

200% of the baseline creatinine value.

**ICU-free days** – Intensive care unit-free days to day 28 (ICU-free days) will be defined as the number of days from the time of the patient’s physical transfer out of the ICU until day 28 after enrollment. Patients who die prior to day 28 after enrollment will receive a value of 0 for ICU-free days. Patients who are never transferred out of the ICU prior to day 28 after enrollment will receive a value of 0 for ICU-free days. Patients who are transferred out of the ICU, return to the ICU, and are not subsequently transferred out of the ICU again before day 28 after enrollment will receive a value of 0 for ICU-free days. For patients who are transferred out of the ICU, are readmitted to the ICU, and are subsequently transferred out of the ICU again prior to day 28 after enrollment, ICU-free days will be awarded based on the time of the final transfer out of the ICU prior to day 28 after enrollment.

**Ventilator-free days** – Ventilator-free days to day 28 (VFDs) will be defined as the number of days from the time of initiating unassisted breathing until day 28 after enrollment. Patients who die prior to day 28 after enrollment will receive a value of 0 for VFDs. Patients who never achieve unassisted breathing prior to day 28 after enrollment will receive a value of 0 for VFDs. Patients who achieve unassisted breathing, return to assisted breathing, and do not again achieve unassisted breathing before day 28 after enrollment will receive a value of 0 for VFDs. For patients who achieve unassisted breathing, return to assisted breathing, and subsequently achieve unassisted breathing again prior to day 28 after enrollment, VFDs will be awarded based on the time of the final initiation of unassisted breathing prior to day 28 after enrollment. Survivors who never experience assisted breathing will receive 28 VFDs.

**Vasopressor-free days** – Vasopressor-free days to day 28 will be defined as the number of days from the time of vasopressor cessation until day 28 after enrollment. Patients who die prior to day 28 after enrollment will receive a value of 0 for vasopressor-free days. Patients who never cease to receive vasopressors prior to day 28 after enrollment will receive a value of 0 for vasopressor-free days. Patients who achieve vasopressor cessation, return to receiving vasopressors, and do not again achieve vasopressor cessation before day 28 after enrollment will receive a value of 0 for vasopressor-free days. For patients who achieve vasopressor cessation, return to receiving vasopressors, and subsequently achieve cessation of vasopressors again prior to day 28 after enrollment, vasopressor-free days will be awarded based on the time of the final cessation of vasopressors prior to day 28 after enrollment. Survivors who never receive vasopressors will receive 28 vasopressor-free days.

**Renal replacement therapy-free days** – Renal replacement therapy-free days to day 28 (RRT-free days) will be defined as the number of days from the time the final RRT treatment until day 28 after enrollment. Patients who die prior to day 28 after enrollment will receive a value of 0 for RRT-free days. Patients who continue to receive RRT through day 28 after enrollment will receive a value of 0 for RRT-free days. Patients who achieve RRT cessation, return to receiving RRT, and do not again achieve RRT cessation before day 28 after enrollment will receive a value of 0 for RRT-free days. For patients who achieve RRT cessation, return to receiving RRT, and subsequently achieve cessation of RRT again prior to day 28 after enrollment, RRT-free days will be awarded based on the time of the final RRT treatment prior to day 28 after enrollment. Survivors who never receive RRT will receive 28 RRT-free days.

**B. Electronic Health Record-based Data Collection**

**Electronically-extracted data –** Structured data from the study institution’s enterprise EHR will be exported daily to an Enterprise Data Warehouse (EDW), along with data from the patient registration, billing, and laboratory clinical information systems. Patient identifiers (medical record number and encounter number) and a timestamp for study enrollment (date and time of first ICU admission during the hospitalization) will be used to extract the pre- and post-enrollment data elements below^4^.

**Collection of baseline creatinine** – Using all inpatient, outpatient, and emergency department creatinine values from our institutional laboratory clinical information system, we will determine (1) the lowest serum creatinine value between 12 months and 24 h prior to hospital admission, (2) the lowest creatinine value between 24 h prior to hospital admission and the time of ICU admission, and (3) an estimated baseline creatinine value using a previously-described three-variable formula [creatinine =0.74− 0.2 (if female) + 0.08 (if African American) + 0.003 × age (in years)]. A baseline creatinine value for each patient will be determined using the hierarchical approach described above.

**Collection of demographic characteristics** – Gender, age, race, height, weight, and body mass index will be extracted directly from the MediPac patient registration system into the EDW.

**Collection of admitting location** – Admitting location will be extracted directly from the MediPac patient registration system.

**Collection of sepsis diagnosis** – A diagnosis of sepsis or septic shock will be determined according to the criteria outlined by the Centers for Medicare and Medicaid Services and the National Center for Health Statistics in the International Classification of Diseases, 10th Edition, Clinical Modification System (ICD-10-CM) Official Guidelines for Coding and Reporting^5^ and the Hospital Inpatient Quality Reporting Program Measures ICD-10-CM DRAFT Code Sets^6^. Sepsis or septic shock will be considered to be present if billing records for the hospitalization contain, in the first five billing codes, any of the following ICD-10-CM codes: A02.1, A22.7, A26.7, A32.7, A40.0, A40.1, A40.3, A40.8, A40.9, A41.01, A41.02, A41.1, A41.2, A41.3, A41.4, A41.50, A41.51, A41.52, A41.53, A41.59, A41.81, A41.89, A41.9, A42.7, A54.86, B37.7, R65.20, R65.21; or any of the corresponding ICD-9-CM codes: 038.0, 038.1, 038.11, 038.12, 038.19, 038.2, 038.3, 038.4, 038.41, 038.42, 038.43, 038.44, 038.49, 038.8, 038.9, 995.91, 995.92.

**Collection of traumatic brain injury diagnosis –** A diagnosis of traumatic brain injury will be determined according to the proposed ICD-10-CM surveillance definition for traumatic brain injury outlined by the National Center for Health Statistics and the National Center for Injury Prevention and Control^7^. Traumatic brain injury will be considered to be present if billing records for the hospitalization contain, in the first five billing codes, any of the following ICD-10-CM codes: S02.0, S02.1-, S02.8, S02.91, S04.02, S04.03-, S04.04-, S06-, S07.1 (code T74.4 shaken infant syndrome, not included due to exclusion of patients less than 18 years old).

**Collection of severity of illness** – Our institution participates in the University HealthSystem Consortium and routinely obtains their All Hospitals Clinical Database, which provides an estimated mortality for each inpatient encounter based on coded data for age, gender, comorbidities, admission source, race, and principal diagnosis. These mortality estimates are based on Medicare Severity-Diagnosis Related Groupings (details at www.uhc.edu). We will retrieve these mortality estimates via our EDW for each patient in our cohort.

**Receipt of intravenous crystalloids** – We have created a list of intravenous crystalloid fluids by manual review of all of the fluid administration types administered at our hospital. This list will be used to extract fluid administration data from our nursing flowsheet in Horizon Expert Documentation, via our EDW. We will retrieve data by matching against medical record number and date of administration.

**Receipt of other fluids** – Data on non-crystalloid fluid administration will be extracted from our nursing flowsheets using the process described above.

**Receipt of blood products** – Data on blood product administration will be extracted from our nursing flowsheets using the process described above.

**Serum laboratory values** – We will create a list of applicable labs by manual review of all laboratory types which match the values of interest, and extract laboratory values from our Cerner laboratory system via our EDW.

**Receipt of renal replacement therapy** – RRT will be identified electronically by the presence of any one of the following American Medical Association’s Current Procedural Terminology (CPT) codes (3066 F, 4054 F, 4055 F, 90963, 90964, 90965, 90966, 90967, 90968, 90969, 90970, 90989, 90993, G0257, G8714, G8956, G9013, G9014, G9231, 90935, 90937, 90945, 90947, 90989, 90993, 90921, 90925, 90999) or International Classification of Disease, Clinical Modification (ICD) codes for ICD-9 (39.95, 54.98) and ICD-10 (5A1D00Z, 5A1D60Z, 3E1M39Z) in the patient registration system or billing system^4^.

**New receipt of renal replacement therapy** – For all patients identified as receiving RRT during the study period, a full text search of the pre-enrollment record will be performed manually by study personnel using terms related to receipt of RRT to identify patients who had received RRT prior to enrollment at an outside facility. Search terms will include “renal replacement’, “RRT”, “CRRT”, “dialysis”, “HD”, “PD”, “end-stage renal”, and “ESRD”. Patients who have not received RRT prior to enrollment and receive RRT between enrollment and hospital discharge, censored at 30 days, will be considered to have met the “new receipt of renal replacement therapy” component of the MAKE30 endpoint^4^.

**Receipt of mechanical ventilation** – Mechanical ventilation will be determined by review of Medipac technical billing data. The number of calendar days with billing for mechanical ventilation will be retrieved for each continuous period that each patient is admitted to an intensive care unit.

**Receipt of vasopressors** – Administration of vasopressors will be determined by review of Medipac technical billing data. The number of calendar days with billing for vasopressors will be retrieved for each continuous period that each patient is admitted to an intensive care unit.

**In-hospital mortality** – In-hospital mortality will be determined by searching for a mortality-associated discharge disposition in our patient registration system after the date of enrollment. Patients with a mortality-associated discharge disposition within 30 days of study enrollment will be considered to have met the mortality component of the MAKE30 endpoint^4^ and those with a mortality-associated discharge disposition within 60 days of study enrollment will be considered to have met criteria for the secondary endpoint of 60-day in-hospital mortality.

**C. Power Calculation**

***Sample Size (Initial Protocol 8/21/14)***

*In clinical practice, the use of balanced intravenous fluids instead of chloride-rich fluids is an intervention with no increased cost and both types of fluids are equally available to the practitioner. Therefore any difference between treatment groups is clinically meaningful in regards to the MAKE30 primary endpoint. In previous studies using the MAKE30 composite endpoint in critically ill patients, the development of this endpoint occurred at a rate of 22%. SMART-MED is anticipated to enroll between 3,000 and 3,600 patients over the one year study period. Barring logistical difficulties, SMART-SURG is anticipated to enroll between 5,000 and 6,500 patients over the one year study period. Enrollment of 8,000 patients in the SMART study overall would allow detection of a difference of 2.6% in the incidence of the primary endpoint with 80% power using a type I error of 0.05.*

**Revised Sample Size Calculation (Protocol Revision 5/10/16)**

The initial protocol called for a study duration of 12 months in 5 units (60 unit-months) based on an anticipated MAKE30 event rate of 22.0%. This event rate was based on prior observational data^8^, but was not specific to the study institution. We have subsequently conducted a pilot trial in the medical ICU and examined a small amount of observational data regarding the incidence of MAKE30 in the non-medical ICUs. These data suggest (1) variation in the rate of MAKE30 between ICUs and (2) an overall rate of MAKE30 in the range of 15-17%, lower than anticipated in our initial power calculation. In order to retain adequate power to detect a relative risk reduction in the initially specified range, we plan to increase the duration of the study to include a total of 82 unit-months over a calendar period of two years. The total number of patients enrolled will depend on the rates of admissions to study ICUs during the fixed time period of the trial, however, we anticipate at least 12,000 patients to be enrolled under the revised study duration.

**Detectable Difference between Groups with the Anticipated Sample Size**

In preparation for SMART, we electronically collected the same dataset that would be used to analyze SMART from the records of all 11,582 patients admitted to the study ICUs between January 1, 2014 and December 31, 2014 – the calendar year prior to the initiation of SMART (approved by the Vanderbilt Institutional Review Board as part of preparation for research). When we analyzed the data from the year prior assuming an alternating sequence of one-month group assignments within each ICU, the point estimate and 95% confidence interval for the odds of MAKE30 comparing one simulated group assignment to the other were numerically identical when derived from a simple chi-square test compared with a generalized linear mixed effect model or generalized estimating equations accounting for cluster and period effects. Thus, for simplicity, we present estimates of the anticipated detectable difference in MAKE30 between arms in SMART using an unadjusted Fisher’s exact test over the potential ranges of possible rates of MAKE30 in the saline group and total number of patients enrolled (table below).

**Based on data from the year prior we anticipate the total enrollment in SMART to be around 14,000 and the overall rate of MAKE30 to be around 15%, resulting in a detectible absolute risk reduction of 1.9% or relative risk reduction of 12%.**  The largest prior trial of saline versus balanced crystalloids reported a point estimate for the relative risk reduction in favor of balanced crystalloids of 12%^9^.

| **Total enrollment** | **Power** | **Alpha** | **MAKE30 saline group** | **Detectable Difference** |
| --- | --- | --- | --- | --- |
| 10,000 | 90% | 0.05 | 17.0% | 2.4% |
| 11,000 | 90% | 0.05 | 17.0% | 2.3% |
| 12,000 | 90% | 0.05 | 17.0% | 2.2% |
| 13,000 | 90% | 0.05 | 17.0% | 2.1% |
| 14,000 | 90% | 0.05 | 17.0% | 2.0% |
| 15,000 | 90% | 0.05 | 17.0% | 2.0% |
| 16,000 | 90% | 0.05 | 17.0% | 1.9% |
|  |  |  |  |  |
| 10,000 | 90% | 0.05 | 16.0% | 2.3% |
| 11,000 | 90% | 0.05 | 16.0% | 2.2% |
| 12,000 | 90% | 0.05 | 16.0% | 2.1% |
| 13,000 | 90% | 0.05 | 16.0% | 2.0% |
| 14,000 | 90% | 0.05 | 16.0% | 2.0% |
| 15,000 | 90% | 0.05 | 16.0% | 1.9% |
| 16,000 | 90% | 0.05 | 16.0% | 1.8% |
|  |  |  |  |  |
| 10,000 | 90% | 0.05 | 15.0% | 2.3% |
| 11,000 | 90% | 0.05 | 15.0% | 2.2% |
| 12,000 | 90% | 0.05 | 15.0% | 2.1% |
| 13,000 | 90% | 0.05 | 15.0% | 2.0% |
| **14,000** | **90%** | **0.05** | **15.0%** | **1.9%** |
| 15,000 | 90% | 0.05 | 15.0% | 1.9% |
| 16,000 | 90% | 0.05 | 15.0% | 1.8% |
|  |  |  |  |  |
| 10,000 | 90% | 0.05 | 14.0% | 2.2% |
| 11,000 | 90% | 0.05 | 14.0% | 2.1% |
| 12,000 | 90% | 0.05 | 14.0% | 2.0% |
| 13,000 | 90% | 0.05 | 14.0% | 1.9% |
| 14,000 | 90% | 0.05 | 14.0% | 1.9% |
| 15,000 | 90% | 0.05 | 14.0% | 1.8% |
| 16,000 | 90% | 0.05 | 14.0% | 1.7% |

**D. Development of the Model for the Primary Analysis**

In preparation for SMART, we electronically collected the same set of variables that would be used to analyze SMART from the records of all 11,582 patients admitted to the study ICUs between January 1, 2014 and December 31, 2014. With the data on MAKE30 from the 11,582 patients admitted in the year before the trial, using generalized linear mixed-effects modeling treating the five ICUs as clusters and the 12 months as periods, we calculated the intra-cluster correlation coefficient to be 0.142, the intra-period correlation coefficient to be 0.026, and the intra-cluster intra-period correlation coefficient to be <0.001.

Using the data from the 11,582 patients admitted in the year before the trial, we performed a series of simulated trials evaluating a variety of trial conditions and potential results including: (1) outcome differences between groups ranging from no difference to a 50% relative risk reduction, (2) heterogeneity of treatment effect by cluster, (3) change in the incidence of the outcome in the control group over the course of the trial, (4) varying levels of intra-cluster correlation, and (5) varying levels of intra-period correlation. In each case the generalized linear mixed-effect model appeared to adequately account for the correlation between participants. Analysis using generalized linear mixed-effects modeling and generalized estimating equations accounting for the same variables produced identical odds ratios and 95% confidence intervals for the difference between the simulated groups in the primary outcome.

**E. Interim Analyses**

***Interim Analysis (Initial Protocol 8/22/14)***

*Enrollment will occur over an expected one year period in which all Vanderbilt ICUs are randomly assigned to one month blocks of alternating balanced fluids only or 0.9% saline only. Blocks will be only one month in length to minimize the effect of seasonal variability.*

*Thirty days after the conclusion of the sixth month of the study, the DSMB will review one interim analysis to determine if further study is warranted. The stopping boundary for efficacy will be met if (1) the difference in the incidence of the primary outcome (MAKE30) between groups is greater than or equal to 2.6% with a p value less than 0.001 AND (2) the p value is less than 0.001 for either death or new renal replacement therapy. As even small differences between groups would be clinically meaningful and given the importance to determine with as much certainty as possible whether balanced fluids are superior to chloride-rich fluids, there will not be a futility stopping boundary.*

**Interim Analyses (Protocol Revision 5/10/16)**

With the initial trial duration scheduled for 12 months in 5 ICUs (60 ICU-months), we planned for a single interim analysis by the DSMB 6 months after trial initiation. As part of the amendment to increase the duration of the study to 82 ICU-months over almost two calendar years, the DSMB will conduct a second interim analysis midway between the initial interim analysis and the revised study stop date. This second interim analysis will include all patients enrolled through July 31st 2016 and will use the same stopping criteria as used the first interim analysis. Specifically, the stopping boundary for efficacy will be met if (1) the difference in the incidence of the primary outcome (MAKE30) between groups is greater than or equal to 2.6% with a p value less than 0.001 AND (2) the p value is less than 0.001 for either death or new renal replacement therapy. As even small differences between groups would be clinically meaningful and given the importance to determine with as much certainty as possible whether balanced fluids are superior to chloride-rich fluids, there will not be a futility stopping boundary.

**F. Handling of Missing Baseline Creatinine**

For patients without a measured serum creatinine between 12 months prior to hospital admission and enrollment, baseline creatinine value for the primary analysis will be estimated using a previously-described three-variable formula [creatinine = 0.74 − 0.2 (if female) + 0.08 (if African American) + 0.003 × age (in years)]^1^. Multiple sensitivity analyses will employ alternative approaches to estimating missing baseline creatinine values:

1. A ‘complete cases’ analysis will be performed in which patients without a measured creatinine value between 12 months prior to hospital admission and enrollment will be excluded.
2. Missing baseline serum creatinine values will be imputed by multivariable single imputation using the R function aregImpute in Hmisc package with 5 imputations. The imputation model will include age, gender, race, group assignment, source of admission, primary diagnosis, receipt of mechanical ventilation, vasopressor receipt, prior hemodialysis, total fluids received in 30 days, UHC expected mortality, overall mortality, new RRT received, minimum creatinine value, maximum creatinine value, and final study creatinine value. Continuous variables will be transformed via cubic splines with 3 to 5 knots.
3. Simple imputation will be performed in which first serum creatinine value after enrollment is used as the baseline creatinine.
4. Simple imputation will be performed in which the highest serum creatinine value between enrollment and 30 days is used as the baseline creatinine.
5. Simple imputation will be performed in which the lowest serum creatinine value between enrollment and 30 days is used as the baseline creatinine.

**SUPPLEMENTAL TABLES**

**Table S1. Composition of the study fluids.**

|  | **Sodium** | **Potassium** | **Calcium** | **Magnesium** | **Chloride** | **Acetate** | **Lactate** | **Gluconate** | **Osmolarity** |
| --- | --- | --- | --- | --- | --- | --- | --- | --- | --- |
| Plasma | 135–145 | 4.5–5.0 | 2.2–2.6 | 0.8–1.0 | 94–111 |  | 1–2 |  | 275–295 |
| 0.9% saline | 154 |  |  |  | 154 |  |  |  | 308 |
| Lactated Ringer’s | 130 | 4.0 | 2.7 |  | 109 |  | 28 |  | 273 |
| Plasma-Lyte A® | 140 | 5.0 |  | 3.0 | 98 | 27 |  | 23 | 294 |

All values are in mEq/L except calculated osmolarity, which is in mOsm/L. 0.9% saline is “Sodium Chloride Injection, USP”, Lactated Ringer’s is “Lactated Ringer’s Injection, USP”, and Plasma-Lyte A® is “Multiple Electrolyte Injection, Type 1, USP”, all from Baxter Healthcare Corporation in Deerfield, IL, USA.

**Table S2. Elixhauser comorbidity index.**

|  | **Saline** | **Balanced** |
| --- | --- | --- |
| **Comorbidity, No. (%)** | **(n = )** | **(n = )** |
| Congestive heart failure | -- | -- |
| Cardiac arrhythmias | -- | -- |
| Valvular disease | -- | -- |
| Pulmonary circulation disorders | -- | -- |
| Hypertension, uncomplicated | -- | -- |
| Hypertension, complicated | -- | -- |
| Paralysis | -- | -- |
| Other neurological disorders | -- | -- |
| Chronic pulmonary disease | -- | -- |
| Diabetes, uncomplicated | -- | -- |
| Diabetes, complicated | -- | -- |
| Hypothyroidism | -- | -- |
| Renal failure | -- | -- |
| Liver disease | -- | -- |
| Peptic ulcer disease excluding bleeding | -- | -- |
| Acquired immunodeficiency syndrome | -- | -- |
| Lymphoma | -- | -- |
| Metastatic cancer | -- | -- |
| Solid tumor without metastasis | -- | -- |
| Rheumatoid arthritis / collagen vascular disease | -- | -- |
| Coagulopathy | -- | -- |
| Obesity | -- | -- |
| Weight loss | -- | -- |
| Fluid and electrolyte disorders | -- | -- |
| Blood loss anemia | -- | -- |
| Deficiency anemias | -- | -- |
| Alcohol abuse | -- | -- |
| Drug abuse | -- | -- |
| Psychoses | -- | -- |
| Depression | -- | -- |

The Elixhauser Comorbidity Index is a method for measuring patient comorbidity based on the International Classification of Diseases (ICD) diagnosis codes (ICD-9-CM and ICD-10) found in administrative data^10,11^.

**Table S3. Intravenous fluids and blood products.**

|  | **Saline** | **Balanced Crystalloid** |  |
| --- | --- | --- | --- |
|  | **(n = )** | **(n = )** | ***P* value** |
| 0.9% sodium chloride, median [IQR]; mean ± SD, mL |  |  |  |
| Prior to enrollment on Day 0 | -- | -- | -- |
| Cumulative volume from enrollment through day 3 | -- | -- | -- |
| Cumulative volume from enrollment through day 7 | -- | -- | -- |
| Cumulative volume from enrollment through day 14 | -- | -- | -- |
| Cumulative volume from enrollment through day 30 | -- | -- | -- |
| Cumulative volume from enrollment through ICU transfer | -- | -- | -- |
| Prior to an ICU crossover in fluid assignment | -- | -- | -- |
| After an ICU crossover in fluid assignment | -- | -- | -- |
| Cumulative volume from ICU transfer to hospital discharge | -- | -- | -- |
| Receipt from enrollment through day 3, No. (%) | -- | -- | -- |
| Receipt from enrollment through day 7, No. (%) | -- | -- | -- |
| Receipt from enrollment through day 14, No. (%) | -- | -- | -- |
| Receipt from enrollment through day 30, No. (%) | -- | -- | -- |
| Receipt from enrollment through ICU transfer, No. (%) | -- | -- | -- |
| Receipt from ICU transfer to hospital discharge, No. (%) | -- | -- | -- |
|  |  |  |  |
| Lactated Ringer’s, median [IQR]; mean ± SD, mL |  |  |  |
| Prior to enrollment on Day 0 | -- | -- | -- |
| Cumulative volume from enrollment through day 3 | -- | -- | -- |
| Cumulative volume from enrollment through day 7 | -- | -- | -- |
| Cumulative volume from enrollment through day 14 | -- | -- | -- |
| Cumulative volume from enrollment through day 30 | -- | -- | -- |
| Cumulative volume from enrollment through ICU transfer | -- | -- | -- |
| Cumulative volume from ICU transfer to hospital discharge | -- | -- | -- |
| Receipt from enrollment through day 3, No. (%) | -- | -- | -- |
| Receipt from enrollment through day 7, No. (%) | -- | -- | -- |
| Receipt from enrollment through day 14, No. (%) | -- | -- | -- |
| Receipt from enrollment through day 30, No. (%) | -- | -- | -- |
| Receipt from enrollment through ICU transfer, No. (%) | -- | -- | -- |
| Receipt from ICU transfer to hospital discharge, No. (%) | -- | -- | -- |
|  |  |  |  |
| Plasma-Lyte A®, median [IQR]; mean ± SD, mL |  |  |  |
| Prior to enrollment on Day 0 | -- | -- | -- |
| Cumulative volume from enrollment through day 3 | -- | -- | -- |
| Cumulative volume from enrollment through day 7 | -- | -- | -- |
| Cumulative volume from enrollment through day 14 | -- | -- | -- |
| Cumulative volume from enrollment through day 30 | -- | -- | -- |
| Cumulative volume from enrollment through ICU transfer | -- | -- | -- |
| Cumulative volume from ICU transfer to hospital discharge | -- | -- | -- |
| Receipt from enrollment through day 3, No. (%) | -- | -- | -- |
| Receipt from enrollment through day 7, No. (%) | -- | -- | -- |
| Receipt from enrollment through day 14, No. (%) | -- | -- | -- |
| Receipt from enrollment through day 30, No. (%) | -- | -- | -- |
| Receipt from enrollment through ICU transfer, No. (%) | -- | -- | -- |
| Receipt from ICU transfer to hospital discharge, No. (%) | -- | -- | -- |
|  |  |  |  |
| Balanced crystalloid, median [IQR]; mean ± SD, mL |  |  |  |
| Prior to enrollment on Day 0 | -- | -- | -- |
| Cumulative volume from enrollment through day 3 | -- | -- | -- |
| Cumulative volume from enrollment through day 7 | -- | -- | -- |
| Cumulative volume from enrollment through day 14 | -- | -- | -- |
| Cumulative volume from enrollment through day 30 | -- | -- | -- |
| Cumulative volume from enrollment through ICU transfer | -- | -- | -- |
| Prior to an ICU crossover in fluid assignment | -- | -- | -- |
| After an ICU crossover in fluid assignment | -- | -- | -- |
| Cumulative volume from ICU transfer to hospital discharge | -- | -- | -- |
| Receipt from enrollment through day 3, No. (%) | -- | -- | -- |
| Receipt from enrollment through day 7, No. (%) | -- | -- | -- |
| Receipt from enrollment through day 14, No. (%) | -- | -- | -- |
| Receipt from enrollment through day 30, No. (%) | -- | -- | -- |
| Receipt from enrollment through ICU transfer, No. (%) | -- | -- | -- |
| Receipt from ICU transfer to hospital discharge, No. (%) | -- | -- | -- |
|  |  |  |  |
| “Isotonic” crystalloid, median [IQR]; mean ± SD, mL |  |  |  |
| Prior to enrollment on Day 0 | -- | -- | -- |
| Cumulative volume from enrollment through day 3 | -- | -- | -- |
| Cumulative volume from enrollment through day 7 | -- | -- | -- |
| Cumulative volume from enrollment through day 14 | -- | -- | -- |
| Cumulative volume from enrollment through day 30 | -- | -- | -- |
| Cumulative volume from enrollment through ICU transfer | -- | -- | -- |
| Cumulative volume from ICU transfer to hospital discharge | -- | -- | -- |
| Receipt from enrollment through day 3, No. (%) | -- | -- | -- |
| Receipt from enrollment through day 7, No. (%) | -- | -- | -- |
| Receipt from enrollment through day 14, No. (%) | -- | -- | -- |
| Receipt from enrollment through day 30, No. (%) | -- | -- | -- |
| Receipt from enrollment through ICU transfer, No. (%) | -- | -- | -- |
| Receipt from ICU transfer to hospital discharge, No. (%) | -- | -- | -- |
|  |  |  |  |
| “Hypotonic” crystalloid, median [IQR]; mean ± SD, mL |  |  |  |
| Prior to enrollment on Day 0 | -- | -- | -- |
| Cumulative volume from enrollment through day 3 | -- | -- | -- |
| Cumulative volume from enrollment through day 7 | -- | -- | -- |
| Cumulative volume from enrollment through day 14 | -- | -- | -- |
| Cumulative volume from enrollment through day 30 | -- | -- | -- |
| Cumulative volume from enrollment through ICU transfer | -- | -- | -- |
| Cumulative volume from ICU transfer to hospital discharge | -- | -- | -- |
| Receipt from enrollment through day 3, No. (%) | -- | -- | -- |
| Receipt from enrollment through day 7, No. (%) | -- | -- | -- |
| Receipt from enrollment through day 14, No. (%) | -- | -- | -- |
| Receipt from enrollment through day 30, No. (%) | -- | -- | -- |
| Receipt from enrollment through ICU transfer, No. (%) | -- | -- | -- |
| Receipt from ICU transfer to hospital discharge, No. (%) | -- | -- | -- |
|  |  |  |  |
| Human albumin solutions, median [IQR]; mean ± SD, mL |  |  |  |
| Prior to enrollment on Day 0 | -- | -- | -- |
| Cumulative volume from enrollment through day 3 | -- | -- | -- |
| Cumulative volume from enrollment through day 7 | -- | -- | -- |
| Cumulative volume from enrollment through day 14 | -- | -- | -- |
| Cumulative volume from enrollment through day 30 | -- | -- | -- |
| Cumulative volume from enrollment through ICU transfer | -- | -- | -- |
| Cumulative volume from ICU transfer to hospital discharge | -- | -- | -- |
| Receipt from enrollment through day 3, No. (%) | -- | -- | -- |
| Receipt from enrollment through day 7, No. (%) | -- | -- | -- |
| Receipt from enrollment through day 14, No. (%) | -- | -- | -- |
| Receipt from enrollment through day 30, No. (%) | -- | -- | -- |
| Receipt from enrollment through ICU transfer, No. (%) | -- | -- | -- |
| Receipt from ICU transfer to hospital discharge, No. (%) | -- | -- | -- |
|  |  |  |  |
| Blood products, median [IQR]; mean ± SD, mL |  |  |  |
| Prior to enrollment on Day 0 | -- | -- | -- |
| Cumulative volume from enrollment through day 3 | -- | -- | -- |
| Cumulative volume from enrollment through day 7 | -- | -- | -- |
| Cumulative volume from enrollment through day 14 | -- | -- | -- |
| Cumulative volume from enrollment through day 30 | -- | -- | -- |
| Cumulative volume from enrollment through ICU transfer | -- | -- | -- |
| Cumulative volume from ICU transfer to hospital discharge | -- | -- | -- |
| Receipt from enrollment through day 3, No. (%) | -- | -- | -- |
| Receipt from enrollment through day 7, No. (%) | -- | -- | -- |
| Receipt from enrollment through day 14, No. (%) | -- | -- | -- |
| Receipt from enrollment through day 30, No. (%) | -- | -- | -- |
| Receipt from enrollment through ICU transfer, No. (%) | -- | -- | -- |
| Receipt from ICU transfer to hospital discharge, No. (%) | -- | -- | -- |
|  |  |  |  |
| Total intravenous fluid, median [IQR]; mean ± SD, mL |  |  |  |
| Prior to enrollment on Day 0 | -- | -- | -- |
| Cumulative volume from enrollment through day 3 | -- | -- | -- |
| Cumulative volume from enrollment through day 7 | -- | -- | -- |
| Cumulative volume from enrollment through day 14 | -- | -- | -- |
| Cumulative volume from enrollment through day 30 | -- | -- | -- |
| Cumulative volume from enrollment through ICU transfer | -- | -- | -- |
| Cumulative volume from ICU transfer to hospital discharge | -- | -- | -- |
| Receipt from enrollment through day 3, No. (%) | -- | -- | -- |
| Receipt from enrollment through day 7, No. (%) | -- | -- | -- |
| Receipt from enrollment through day 14, No. (%) | -- | -- | -- |
| Receipt from enrollment through day 30, No. (%) | -- | -- | -- |
| Receipt from enrollment through ICU transfer, No. (%) | -- | -- | -- |
| Receipt from ICU transfer to hospital discharge, No. (%) | -- | -- | -- |

**Table S4. Serum laboratory values.**

|  | **Saline** | **Balanced** |  |
| --- | --- | --- | --- |
| **Laboratory value** | **(n = )** | **(n = )** | ***P* value** |
| Serum sodium, mmol/L |  |  |  |
| Highest between enrollment and day 30, median [IQR] | -- | -- | -- |
| Lowest between enrollment and day 30, median [IQR] | -- | -- | -- |
| >145 between enrollment and day 30, No. (%) | -- | -- | -- |
| < 135 between enrollment and day 30, No. (%) | -- | -- | -- |
|  |  |  |  |
| Serum potassium, mmol/L |  |  |  |
| Highest between enrollment and day 30, median [IQR] | -- | -- | -- |
| Lowest between enrollment and day 30, median [IQR] | -- | -- | -- |
| > 5.0 between enrollment and day 30, No. (%) | -- | -- | -- |
| < 3.0 between enrollment and day 30, No. (%) | -- | -- | -- |
|  |  |  |  |
| Serum chloride, mmol/L |  |  |  |
| Highest between enrollment and day 30, median [IQR] | -- | -- | -- |
| Lowest between enrollment and day 30, median [IQR] | -- | -- | -- |
| > 110 between enrollment and day 30, No. (%) | -- | -- | -- |
| < 90 between enrollment and day 30, No. (%) | -- | -- | -- |
|  |  |  |  |
| Serum bicarbonate, mmol/L |  |  |  |
| Highest between enrollment and day 30, median [IQR] | -- | -- | -- |
| Lowest between enrollment and day 30, median [IQR] | -- | -- | -- |
| > 30 between enrollment and day 30, No. (%) | -- | -- | -- |
| < 20 between enrollment and day 30, No. (%) | -- | -- | -- |

**Table S6. Indications for new renal replacement therapy.**

|  | **Saline** | **Balanced** |  |
| --- | --- | --- | --- |
| **Indication*, No. (%)** | **(n = )** | **(n = )** | ***P* Value** |
| Oliguria | -- | -- | -- |
| Hyperkalemia with serum potassium > 6.5 mEq/L | -- | -- | -- |
| Acidemia with pH < 7.20 | -- | -- | -- |
| Blood urea nitrogen > 70 mg/dL | -- | -- | -- |
| Serum creatinine > 3.39 mg/dL | -- | -- | -- |
| Organ edema | -- | -- | -- |
| Other renal failure–related indication | -- | -- | -- |
| Other non–renal failure–related indication | -- | -- | -- |

* Each patient may have more than one.

**SUPPLEMENTAL REFERENCES**

1. Závada J, Hoste E, Cartin-Ceba R, et al. A comparison of three methods to estimate baseline creatinine for RIFLE classification. *Nephrol Dial Transplant Off Publ Eur Dial Transpl Assoc - Eur Ren Assoc*. 2010;25(12):3911-3918. doi:10.1093/ndt/gfp766.

2. Kidney Disease: Improving Global Outcomes (KDIGO) Acute Kidney Injury Work Group. KDIGO Clinical Practice Guideline for Acute Kidney Injury. *Kidney inter*. 2012;2(Suppl):1–138.

3. Levey AS, Stevens LA, Schmid CH, et al. A new equation to estimate glomerular filtration rate. *Ann Intern Med*. 2009;150(9):604-612.

4. Semler MW, Rice TW, Shaw AD, et al. Identification of Major Adverse Kidney Events Within the Electronic Health Record. *J Med Syst*. 2016;40(7):167. doi:10.1007/s10916-016-0528-z.

5. ICD-10-CM Official Guidelines for Coding and Reporting. FY 2016. https://www.cms.gov/medicare/coding/icd10/downloads/2016-icd-10-cm-guidelines.pdf. Accessed August 4, 2016.

6. Centers for Medicare and Medicaid Services (CMS) and the National Center for Health Statistics (NCHS). Hospital Inpatient Quality Reporting Program Measures International Classification of Diseases, 10th Edition, Clinical Modification System (ICD-10-CM) DRAFT Code Sets. FY 2016. https://www.cms.gov/Medicare/Quality-Initiatives-Patient-Assessment-Instruments/HospitalQualityInits/Downloads/HIQR-ICD9-to-ICD10-Tables.pdf. Accessed August 4, 2016.

7. Hedegaard H, Johnson RL, Warner M, Chen L-H, Annest JL. Proposed Framework for Presenting Injury Data Using the International Classification of Diseases, Tenth Revision, Clinical Modification (ICD-10-CM) Diagnosis Codes. *Natl Health Stat Rep*. 2016;(89):1-20.

8. Kashani K, Al-Khafaji A, Ardiles T, et al. Discovery and validation of cell cycle arrest biomarkers in human acute kidney injury. *Crit Care Lond Engl*. 2013;17(1):R25. doi:10.1186/cc12503.

9. Young P, Bailey M, Beasley R, et al. Effect of a Buffered Crystalloid Solution vs Saline on Acute Kidney Injury Among Patients in the Intensive Care Unit: The SPLIT Randomized Clinical Trial. *JAMA*. October 2015:1-10. doi:10.1001/jama.2015.12334.

10. Elixhauser A, Steiner C, Harris DR, Coffey RM. Comorbidity measures for use with administrative data. *Med Care*. 1998;36(1):8-27.

11. Quan H, Sundararajan V, Halfon P, et al. Coding algorithms for defining comorbidities in ICD-9-CM and ICD-10 administrative data. *Med Care*. 2005;43(11):1130-1139.
