# Supplementary material for: Balanced crystalloids versus saline in the intensive care unit: study protocol for a cluster-randomized, multiple-crossover trial
Source: Trials. 2017 Mar 16;18:129. doi: 10.1186/s13063-017-1871-1 (PMC5356286; doi:10.1186/s13063-017-1871-1)
Supplement: Additional file 3: — This file contains a .pdf version of the R code that will be used to analyze the final study data. (PDF 118 kb) [file 13063_2017_1871_MOESM3_ESM.pdf]

Study Protocol and Statistical Analysis Plan for the Isotonic Solutions and Major Adverse Renal Events Trial (SMART)

Li Wang and Daniel Byrne
Department of Biostatistics
Vanderbilt University School of Medicine

December 9, 2016

1 Tables

2 Figures

3 Primary Analysis

4 Sensitivity Analysis - Missing Creatinine Measures

A All R Code

```
# Load R packages
require(rms)
require(lme4)
require(knitr)

knitrSet(echo=TRUE,cache=FALSE)
ltx <- function(x, ...) {
  if('caption' %in% names(list(...))) latex(x, ..., file='')
  else latex(x, table.env=FALSE, ..., file='')
}

# Set working directory
setwd("")
# Read data into R
load("SMART.fulldata.R")
# Load R functions
source("LoessWithCI.R")
source("reshape.R")
source("SummaryTable.R")

# Table 1 - Comparisons of baseline characteristics between study groups
options(digits=3)
ltx(summaryM(unit + patient.age + patient.gender + patient.race.v2 + patient.weight + patient.bmi +
             source.of.admission + primary.diagnosis + Sepsis + TBI + ventilator.flag + inotropic.flag +
             uhc.expected.mort + hemodialysis.pre.icu + lowestcreatpriortoenc + mostrecentcreatpriortoenc +
             lowestcreatdurencpriorto.icu + lowestcreatdurencpriorto.icu.imp + make30.baselinecrsource +
             make30.baselinecr + make30.baselinecr.calculate + make30.baselinecr.DurEncPriorToICU +
             make30.baselinecr.PriorToEnc + present.AKI + present.CKD +  present.CKD.calculate +
             present.CKD.DurEncPriorToICU + present.CKD.PriorToEnc ~ group, data = fulldata, test=TRUE),
    where="htbp", caption="Patient characteristics at baseline", exclude1 = FALSE, prN=TRUE, prn=FALSE,
    long = TRUE, prmsd = TRUE, npct = 'both', prtest='P', size="footnotesize", digits=2, longtable=TRUE,
    landscape=FALSE, cener="centering", label = 'table:basedesc', what = '%', pctdig=3,
    colheads = c('NS','BL','Test statistics'), lines.page=62, rowlabel.just = "p{0.25\\linewidth}")

# Table 2 - Comparison of patients' outcomes between groups
ltx(summaryM(make30.final + make30.criteriamet + make30.death + new.rrt.verified + make30.crrt +
             make30.renaldysfunction.persistent +  make30.renaldysfunction.persistent.survivor +
             make30.renaldysfunction.persistent.survivor.woRRT + make30.renaldysfunction.kdigo +
             died.in.ICU + died.30day + died.60day + icufreeday + ventfreeday + inotropicfreeday +
             rrtfreeday + rrtday + ventilator.flag + unit.ventilator.days + inotropic.flag +
             unit.inotropic.days + make30.maxcr + make30.baselinecr + make30.finalcreatbeforedischarge +
             make30.finalstudycreat + make30.finalstudycreat.survivor + highestdailycreat.max +
             change.creat + deathinicu + incidentAKI ~ group, data = fulldata, test=TRUE, continuous=3),
    where="htbp", caption="Patient outcomes", exclude1 = FALSE, prN=TRUE,prn=FALSE,
    long = TRUE, prmsd = TRUE, npct = 'both', prtest='P', size="footnotesize", digits=2, longtable=TRUE,
    landscape=FALSE, cener="centering", label = 'table:outcome', what = '%', pctdig=3,
    colheads = c('NS','BL','Test statistics'), lines.page=62, rowlabel.just = "p{0.25\\linewidth}")

## We will present the unadjusted effect and the effect adjusted for cluster and period for all these
## secondary outcomes

# Comparison of IV fluids received between groups - supplemental table
temp <- expand.grid(c('ns.base','lr','plasmalyte','balanced','isotonic.crystalloid',
                      'nonisotonic.crystalloid','albumins','bloodproduct','total.fluid'),
                    c('b',0:7,'cum3','cum7','cum14','cum30','unit','unitbeforecross','unitaftercross','hosp'))
varList <- apply(temp[order(temp[,1]),], 1, paste, collapse=".")
```

```r
ltx(summaryM(as.formula(paste(paste(varList, collapse=" + "), "group", sep=" ~ ")),
             data = fulldata, test=TRUE, continuous=4),
    where="htbp", caption="Fluids", exclude1 = FALSE, prN=TRUE, prn=FALSE, vnames="name",
    long = TRUE, prmsd = TRUE, npct = 'both', prtest='P', size="footnotesize", digits=2,
    longtable=TRUE, landscape=FALSE, cener="centering", label = 'table:fluid', what = '%', pctdig=3,
    colheads = c('NS','BL','Test statistics'), lines.page=66, rowlabel.just = "p{0.2\\linewidth}")

# Comparison of IV fluids received between groups - supplemental table
temp <- expand.grid(c('ns.base','lr','plasmalyte','balanced','isotonic.crystalloid',
                      'nonisotonic.crystalloid','albumins','bloodproduct','total.fluid'),
                    paste0(c('b',0:7,'cum3','cum7','cum14','cum30','unit','unitbeforecross',
                             'unitaftercross','hosp'),'_binary'))
varList <- apply(temp[order(temp[,1]),], 1, paste, collapse=".")
ltx(summaryM(as.formula(paste(paste(varList, collapse=" + "), "group", sep=" ~ ")),
             data = fulldata, test=TRUE, continuous=4),
    where="htbp", caption="Fluids", exclude1 = FALSE, prN=TRUE, prn=FALSE, vnames="name",
    long = TRUE, prmsd = TRUE, npct = 'both', prtest='P', size="footnotesize", digits=2, longtable=TRUE,
    landscape=FALSE, cener="centering", label = 'table:fluidbinary', what = '%', pctdig=3,
    colheads = c('NS','BL','Test statistics'), lines.page=66, rowlabel.just = "p{0.2\\linewidth}")

# Comparison of serum laboratory values between groups - supplemental table
temp <- expand.grid(c('firstdaily','lowestdaily','highestdaily'),
                    c('.na.','.k.','.cl.','.co2.','.bun.','creat.'),0:7)
varList <- c(apply(temp[order(temp[,2],temp[,1]),], 1, paste, collapse=""),
             c("lowestdaily.na.min","highestdaily.na.max","na.145","na.135",
               "lowestdaily.k.min","highestdaily.k.max","k.5","k.3",
               "lowestdaily.cl.min","highestdaily.cl.max","cl.110","cl.90" ,
               "lowestdaily.co2.min","highestdaily.co2.max","co2.30","co2.20",
               "lowestdaily.bun.min","highestdaily.bun.max",
               "lowestdailycreat.min","highestdailycreat.max"))
ltx(summaryM(as.formula(paste(paste(varList, collapse=" + "), "group", sep=" ~ ")),
             data = fulldata, test=TRUE,continuous=4),
    where="htbp", caption="Electrolytes", exclude1 = FALSE, prN=TRUE, prn=FALSE,
    long = TRUE, prmsd = TRUE, npct = 'both', prtest='P', size="footnotesize", digits=2, longtable=TRUE,
    landscape=FALSE, cener="centering", label = 'table:elec', what = '%', pctdig=3,
    colheads = c('NS','BL','Test statistics'), lines.page=65, rowlabel.just = "p{0.2\\linewidth}")

# Comprison of indications for new RRT between groups - supplemental table
ltx(summaryM(oliguria + k.6.5 + ph.7.20 + bun.70 + cr.3.39 + organ.edema + other.renal + other.nonrenal +
               rrtuse.postdischarge + text ~ group, data = subset(fulldata, new.rrt.verified==1),
             test=TRUE, continuous=3), where="htbp", caption="Indications for RRT", exclude1 = FALSE,
    prN=TRUE,prn=FALSE, long = TRUE, prmsd = TRUE, npct = 'both', prtest='P', size="small", digits=2,
    longtable=TRUE, landscape=FALSE, cener="centering", label = 'table:indi', what = '%', pctdig=3,
    colheads = c('NS','BL','Test statistics'), lines.page=58, rowlabel.just = "p{0.4\\linewidth}")

# Comparison of baseline comorbidities between groups - supplemental table
ltx(summaryM(cpd + chf + bla + fed + mtc + cvd + cag + dbu + vld + car + peb + aid + lvd + lym +
             pcd + ond + pvo + rnf + obs + wgt + hyu + par + st. + psy + dep + hyc + alc + drg +
             dfa + dbc + thy ~ group, data = fulldata, test=TRUE, continuous=3),
    where="htbp", caption="Elixhauser", exclude1 = FALSE, prN=TRUE,prn=FALSE,
    long = TRUE, prmsd = TRUE, npct = 'both', prtest='P', size="small", digits=2, longtable=TRUE,
    landscape=FALSE, cener="centering", label = 'table:eli', what = '%', pctdig=3,
    colheads = c('NS','BL','Test statistics'), lines.page=57, rowlabel.just = "p{0.2\\linewidth}")
```

```
# Comparison of volume of each study crystaloid received between groups - figure
fig1 <- fulldata[,c('encounternumber','group','ns.base.b','balanced.b',
                    paste0('ns.base.',0:7),paste0('balanced.',0:7))]

fig11<-data.frame(t(with(fig1,apply(fig1[,paste0('ns.base.',0:7)],1,function(x)cumsum(c(x))))))
fig12<-data.frame(t(with(fig1,apply(fig1[,paste0('balanced.',0:7)],1,function(x)cumsum(c(x))))))

names(fig11) <- paste0('ns.base.0.',0:7)
names(fig12) <- paste0('balanced.0.',0:7)

fig1 <- cbind(fig1, fig11, fig12)

fig1.out <- data.frame(day=rep(1:9,4),
                       group=rep(c('salinearm.saline','salinearm.balance',
                                   'balancearm.balance','balancearm.saline'),each=9),
                       mean=rep(NA,36), lower=rep(NA,36), upper=rep(NA,36))

nsL <- c('ns.base.b',paste0('ns.base.0.',0:7))
bL <- c('balanced.b',paste0('balanced.0.',0:7))


for (i in 1:length(nsL)) {
  temp1 <- smean.cl.boot(fig1[[nsL[i]]][fig1$group=='NS'], B=1000, reps=TRUE)
  fig1.out[i,3:5] <- temp1[1:3]
  temp2 <- smean.cl.boot(fig1[[bL[i]]][fig1$group=='NS'], B=1000, reps=TRUE)
  fig1.out[i+9,3:5] <- temp2[1:3]
  temp3 <- smean.cl.boot(fig1[[bL[i]]][fig1$group=='BL'], B=1000, reps=TRUE)
  fig1.out[i+18,3:5] <- temp3[1:3]
  temp4 <- smean.cl.boot(fig1[[nsL[i]]][fig1$group=='BL'], B=1000, reps=TRUE)
  fig1.out[i+27,3:5] <- temp4[1:3]
}

pdf('fig1.pdf', width=8, height=5)
par(mfrow=c(1,2), mar=c(3.5,4,2,1))
plot(1, type="n", axes=F, xlab="", ylab="Cumulative volume (mL)", ylim=c(0,3000),xlim=c(0,9))
with(subset(fig1.out, group=='salinearm.saline'),
     errbar(c(0.7, 2:9), mean, upper, lower, pch=5,cex=1.1,add=TRUE))
with(subset(fig1.out, group=='salinearm.saline' & day > 1), lines(day, mean, lty=2))
with(subset(fig1.out, group=='salinearm.balance'),
     errbar(c(0.3, 2:9), mean, upper, lower, pch=16, cex=1.2, add=TRUE))
with(subset(fig1.out, group=='salinearm.balance' & day > 1), lines(day, mean, lty=1))
legend(0,3100, pch=c(16,5),cex=c(1.2,1.1), c('Balanced fluid','0.9% Saline'), lty=c(1,2), bty='n')
axis(side=1,at=2:9,labels=c('0','1','2','3','4','5','6','7'))
axis(side=2,at=seq(0,3000,by=500), labels=seq(0,3000,by=500))
text(0.6,500, 'Before\nenrollment\non day 0', cex=0.7)
mtext('Saline Arm', side=3, line=0.5, cex=1.3)
mtext('Days after enrollment', side=1, line=2, at=5.5)
lines(c(1.5,1.5), c(0,1500), col = "gray60")
box()

plot(1, type="n", axes=F, xlab="", ylab="Cumulative volume (mL)", ylim=c(0,3000),xlim=c(0,9))
with(subset(fig1.out, group=='balancearm.balance'),
     errbar(c(0.3, 2:9), mean, upper, lower, pch=16, cex=1.2, add=TRUE))
with(subset(fig1.out, group=='balancearm.balance' & day > 1), lines(day, mean, lty=1))
with(subset(fig1.out, group=='balancearm.saline'),
     errbar(c(0.7, 2:9),mean, upper, lower, pch=5,cex=1.1,add=TRUE))
with(subset(fig1.out, group=='balancearm.saline' & day > 1), lines(day, mean, lty=2))
legend(0,3100, pch=c(16,5),cex=c(1.2,1.1), c('Balanced fluid','0.9% Saline'), lty=c(1,2), bty='n')
axis(side=1,at=2:9,labels=c('0','1','2','3','4','5','6','7'))
```

```
axis(side=2,at=seq(0,3000,by=500), labels=seq(0,3000,by=500))
text(0.6, 500, 'Before\nenrollment\non day 0',cex=0.7)
mtext('Balanced Arm', side=3, line=0.5, cex=1.3)
mtext('Days after enrollment', side=1, line=2, at=5.5)
lines(c(1.5,1.5), c(0,1500), col = "gray60")
box()
dev.off()


# Heterogeny of treatment effect - figure
dd <- datadist(fulldata[,c('make30.final','make30.death','new.rrt.verified',
                           'isotonic.crystalloid.cum3','isotonic.crystalloid.cum30',
                           'patient.age','source.of.admission.v2','source.of.admission',
                           'ventilator.flag','inotropic.flag','group','Sepsis','TBI',
                           'hemodialysis.pre.icu','receive.nonassign.crys','uhc.expected.mort',
                           'cat','time','unit')]); options(datadist = 'dd')

NS <- subset(fulldata, group=='NS')
BL <- subset(fulldata, group=='BL')

oout <- vector('list',2)
vvar <- c('unit','source.of.admission.v2','ventilator.flag','inotropic.flag','Sepsis','TBI','cat')
grp <- c('NS','BL')


for (j in 1:2){
  for (i in 1:length(vvar)){
    oout[[j]]  <- c(oout[[j]],paste0(table(fulldata[[vvar[i]]][fulldata$group==grp[j]],
                                           fulldata$make30.final[fulldata$group==grp[j]])[,2],'/',
         table(fulldata[[vvar[i]]][fulldata$group==grp[j]]),'(',
         round(100*prop.table(table(fulldata[[vvar[i]]][fulldata$group==grp[j]],
                                    fulldata$make30.final[fulldata$group==grp[j]]),1)[,2],1),')'))
  }
  oout[[j]] <- c(oout[[j]],
                 paste(
                   paste0(table(fulldata$make30.final[fulldata$group==grp[j]])[2],'/',
                          nrow(subset(fulldata, group==grp[j])),'(',
                          round(100*prop.table(table(fulldata$make30.final[fulldata$group==grp[j]]))[2],
                                1),')')))
}

freq <- paste(oout[[1]], oout[[2]], sep=" ")


vvar <- c('unit','source.of.admission.v2','ventilator.flag','inotropic.flag','Sepsis','TBI','cat')

p.int <- rep(NA, length(vvar))
out <- NA

p.int[1] <- anova(lrm(as.formula(paste("make30.final", paste(c("group",'unit'), collapse="*"),
                                       sep=" ~ ")), data=fulldata, x=TRUE, y=TRUE))[2,3]
for (j in 1:length(levels(fulldata$unit))){
    m.sub <- lrm(make30.final ~ group, data=subset(fulldata,unit==levels(fulldata$unit)[j]), x=TRUE, y=TRUE)
    out <- rbind(out,c(summary(m.sub,group='NS')[2,c(4,6,7)], anova(m.sub)[1,3]))
}
out <- out[-1,]
```

```r
for (i in 2:length(vvar)){
  m.int <- glmer(as.formula(paste("make30.final",
                                paste(paste(c("group",vvar[i]), collapse="*"),"(1 | unit)",sep=" + "),
                                sep=" ~ ")), data=fulldata, family=binomial)
  m.int0 <- glmer(as.formula(paste("make30.final", paste("group",vvar[i], "(1 | unit)",sep=" + "),
                                 sep=" ~ ")), data=fulldata, family=binomial)
  p.int[i] <- anova(m.int, m.int0)$Pr[2]
  for (j in 1:length(levels(fulldata[[vvar[i]]]))){
    m.sub <- glmer(make30.final ~ group + (1 | unit),
                   data=subset(fulldata,fulldata[[vvar[i]]]==levels(fulldata[[vvar[i]]])[j]),
                   family=binomial)
    cc <- confint(m.sub,parm="beta_")
    out <- rbind(out,c(exp(cbind(est=fixef(m.sub),cc)[2,]),
                       summary(m.sub)$coefficients[2,4]))
  }
}

mm <- glmer(make30.final ~ group + (1 | unit), data = fulldata, family = binomial(link=logit))
cc <- confint(mm,parm="beta_")

out <- rbind(out,
             c(exp(cbind(est=fixef(mm),cc)[2,]),
                   summary(mm)$coefficients[2,4]))



# Main secondary analysis: group assignment by crystalloid volume interaction
mo <- lrm(make30.final ~ group * rcs(isotonic.crystalloid.cum30,3), data = fulldata, x = TRUE, y = TRUE)
# Secondary analysis: group assignment by severity of illness interaction
mp <- lrm(make30.final ~ group * uhc.expected.mort, data = fulldata, x = TRUE, y = TRUE)
# Sensitivity analysis: group assignment by crystalloid volume interaction
mq <- lrm(make30.final ~ group * rcs(isotonic.crystalloid.cum3,3), data = fulldata, x = TRUE, y = TRUE)


pdf('fig2.pdf', width=8, height=8)
layout(matrix(c(1,2,3,3,3,3), 3, 2, byrow = TRUE))
fulldata$group2 <- fulldata$group
levels(fulldata$group2) <- c('Saline','Balanced fluid')

par(mar=c(3,4,1,1))
with(fulldata, LoessWithCI(isotonic.crystalloid.cum30,make30.final,group=group, col=c('red','blue'),
                           side=c(3,1), xlim=c(0,10000), ylim=c(0,1), xlab='', ylab='', hists=TRUE,
                           cex.axis=1, textsize=1))
mtext('Total isotonic crystalloid through day 30 (mL)', side=1, line=2.5,cex=1)
mtext('Incidence of MAKE30', side=2, line=2.5,cex=1)
text(10000,0.9,paste0('P-value for interaction = ',sprintf("%4.3f",anova(mo)[2,3])), adj=1)
text(9000,0.33,'Saline',col='red')
text(9000,0.2,'Balanced',col='blue')


par(mar=c(3,4,1,1))
with(fulldata, LoessWithCI(uhc.expected.mort,make30.final,group=group, col=c('red','blue'),
                           side=c(3,1), xlim=c(0,1), ylim=c(0,1), xlab='', ylab='', hists=TRUE,
                           cex.axis=1, textsize=1))
mtext('Predicted in-hospital mortality', side=1, line=2.5,cex=1)
mtext('Incidence of MAKE30', side=2, line=2.5,cex=1)
text(0.65,0.1,paste0('P-value for interaction = ',sprintf("%4.3f",anova(mp)[2,3])))
text(0.85,0.65,'Saline',col='red')
text(0.8,0.9,'Balanced',col='blue')
```

```
par(mar=c(4,28,2,15))
yval <- c(29,28,27,26,25,23,22,21,20,19,17,16,14,13,11,10,8,7,5,4,3,2,1)
hh <- 30:1
yval1 <- hh[-which(30:1 %in% yval)]
OR <- out[,1]
LCI <- out[,2]
UCI <- out[,3]
DIFF <- log(UCI) - log(LCI)
XLAB=''
MAIN=''
L <- length(OR)
AXIS=c(0.1,0.3,1,5,10)
XLIM=c(0.3,2)
PCH=c(rep(16,nrow(out)-1),18)
CEX=c(rep(0.8,nrow(out)-1),2)

plot(OR, yval, xlim = XLIM, pch = PCH, cex = CEX, log = "x", axes = FALSE, xlab = XLAB, ylab = "",
     ylim = c(1, 30), main = MAIN)
points(OR, yval, pch=PCH, cex=CEX)
  for (ii in 1:L){
    if (!is.na(UCI[ii]) & UCI[ii] > XLIM[2]){
      UCI[ii] <- XLIM[2]
    }
  }
  for (ii in 1:L){
    if (!is.na(LCI[ii]) & LCI[ii] < XLIM[1]){
      LCI[ii] <- XLIM[1]
    }
  }
  segments(LCI, yval,UCI, yval, lwd=1)
  arrows(LCI[LCI==XLIM[1]]+0.1, yval[LCI==XLIM[1]], LCI[LCI==XLIM[1]], yval[LCI==XLIM[1]],length=0.06)
  arrows(UCI[UCI==XLIM[2]]-0.1, yval[UCI==XLIM[2]], UCI[UCI==XLIM[2]], yval[UCI==XLIM[2]], length=0.06)
  axis(1, at=AXIS, labels = AXIS, cex=0.5)
  segments(1,0,1,30)

mtext('Favors Balanced',side=1,at=0.6,line=3,cex=0.8)
mtext('Favors Saline',side=1,at=1.7,line=3,cex=0.8)

mtext(c('MICU','CVICU','NEICU','TICU','SICU',
        'Emergency Department','Operating room','Transfer from another hospital','Hospital ward','Other',
        'No','Yes', 'No','Yes', 'No','Yes', 'No','Yes', 'Normal','AKI','CKD','RRT','Overall'),
      side=2, at=yval, line=c(rep(25,length(yval)-1),27),adj=0, cex=c(rep(0.7,length(yval)-1),1),las=2)

mtext(c('Unit','Source of admission','Receipt of mechanical ventilation','Receipt of vasopressors',
        'Sepsis','TBI','Categories of kidney function'), side=2, at=yval1, line=27,adj=0, cex=0.7,las=2)
mtext(oout[[1]], at=yval, side=2, line=7, adj=1, las=2, cex=0.7)
mtext(oout[[2]], at=yval, side=2, line=0, adj=1, las=2, cex=0.7)
mtext('Saline',at=31, side=2, line=7, adj=1, las=2, cex=1)
mtext('Balanced',at=31, side=2, line=0, adj=1, las=2, cex=1)
arrows(0.8, -1.5, 0.5, -1.5, length=0.1, xpd = TRUE)
arrows(1.3, -1.5, 2, -1.5, length=0.1, xpd = TRUE)
mtext(paste0(sprintf("%3.2f",out[,1]),'(',sprintf("%3.2f",out[,2]),'-',sprintf("%3.2f",out[,3]),')'),
      at=yval, side=4, line=0,adj=0,las=2,cex=0.7)
mtext(sprintf("%4.3f",out[,4]),at=yval, side=4, line=7, adj=0, las=2, cex=0.7)
mtext(sprintf("%4.3f",p.int),at=yval1, side=4, line=12, adj=0, las=2, cex=0.7)
mtext('P Value', at=31, side=4, line=6, adj=0, las=2, cex=0.7)
mtext('P Value for', at=31.5, side=4, line=10, adj=0, las=2, cex=0.7)
mtext('Interaction', at=31, side=4, line=10, adj=0, las=2, cex=0.7)
```

```
mtext('Odds Ratio (95% CI)', side=3, line=-0.5, cex=1)
dev.off()


# Comparison of first, lowest, and highest serum laboratory value each day between groups - figure
elec.long=table.to.long(fulldata,
                        list(firstdaily.na=paste0("firstdaily.na.",0:7),
                             lowestdaily.na=paste0("lowestdaily.na.",0:7),
                             highestdaily.na=paste0("highestdaily.na.",0:7),

                             firstdaily.k=paste0("firstdaily.k.",0:7),
                             lowestdaily.k=paste0("lowestdaily.k.",0:7),
                             highestdaily.k=paste0("highestdaily.k.",0:7),

                             firstdaily.cl=paste0("firstdaily.cl.",0:7),
                             lowestdaily.cl=paste0("lowestdaily.cl.",0:7),
                             highestdaily.cl=paste0("highestdaily.cl.",0:7),

                             firstdaily.co2=paste0("firstdaily.co2.",0:7),
                             lowestdaily.co2=paste0("lowestdaily.co2.",0:7),
                             highestdaily.co2=paste0("highestdaily.co2.",0:7),

                             firstdaily.bun=paste0("firstdaily.bun.",0:7),
                             lowestdaily.bun=paste0("lowestdaily.bun.",0:7),
                             highestdaily.bun=paste0("highestdaily.bun.",0:7),

                             firstdaily.creat=paste0("firstdailycreat.",0:7),
                             lowestdaily.creat=paste0("lowestdailycreat.",0:7),
                             highestdaily.creat=paste0("highestdailycreat.",0:7)))
elec.long <- elec.long[,c("encounternumber","group","firstdaily.na.score","lowestdaily.na.score",
                          "highestdaily.na.score","firstdaily.k.score","lowestdaily.k.score",
                          "highestdaily.k.score","firstdaily.cl.score","lowestdaily.cl.score",
                          "highestdaily.cl.score","firstdaily.co2.score","lowestdaily.co2.score",
                          "highestdaily.co2.score","firstdaily.bun.score","lowestdaily.bun.score",
                          "highestdaily.bun.score","firstdaily.creat.score","lowestdaily.creat.score",
                          "highestdaily.creat.score")]
elec.long$day=rep(0:7,nrow(fulldata))

dd.eleclong <- datadist(elec.long); options(datadist = 'dd.eleclong')


elec.model.list1 <- c('first','lowest','highest')
elec.model.list2 <- c('na','k','cl','co2','bun','creat')
ylablist <- c('Sodium (mmol/L)','Potassium (mmol/L)','Chloride (mmol/L)',
              'Bicarbonate (mmol/L)','Blood urea nitrogen (mg/dL)','Creatinine (mg/dL)')
pval <- matrix(rep(NA,3*6*2), ncol=12)

for (i in 1:length(elec.model.list1)){
  for (j in 1:length(elec.model.list2)){
    form <- as.formula(paste(paste0(elec.model.list1[i],'daily.',elec.model.list2[j],'.score'),
                             'group*rcs(day,3)', sep=' ~ '))
    tempmod <- robcov(lrm(form, data=elec.long, x = TRUE, y = TRUE), cluster = elec.long$encounternumber)
    pval[i,(j-1)*2+1] <- sprintf("%4.3f", anova(tempmod)[1,3])
    pval[i,(j-1)*2+2] <- sprintf("%4.3f", anova(tempmod)[2,3])
  }
}
pval0 <- pval
pval <- matrix(ifelse(as.numeric(pval0)<0.001,'<.001',pval0),ncol=12)
```

```
for (i in 1:3){
  pdf(paste0('fig',i+2,'.pdf'), width=8, height=6)
  layout(matrix(c(1,2,3,4,5,6), ncol=3))
  par(mar=c(3.5,4,1,1))
  outcomelist <- paste0(elec.model.list1[i],'daily.',elec.model.list2,'.score')
  for (j in 1:6){
    LoessWithCI(elec.long$day, elec.long[[outcomelist[j]]], group=elec.long$group,
             col=c('red','blue'), side=c(3,1), pprint=TRUE,
             pval1=pval[i,(j-1)*2+1], pval2=pval[i,(j-1)*2+2],
             xlab='Day', ylab=ylablist[j],
             cex.axis=1.5, textsize=1.5, curvelabprint=TRUE,
             labellab=c('Saline','Balanced'), labelcol=c('red','blue'))
  }
  dev.off()
}


# Sensitivity analysis of interaction between study groups and crystalloid received through day 3
# supplemental figure
pdf("fig6.pdf")
par(mfrow=c(1,1),mar=c(4,4,1,2))
with(fulldata, LoessWithCI(isotonic.crystalloid.cum3,make30.final,group=group, col=c('red','blue'),
                           side=c(3,1), xlim=c(0,10000), ylim=c(0,1), xlab='', ylab='', hists=TRUE,
                           cex.axis=1.5, textsize=1.5, curvelabprint=TRUE,
                           labellab=c('Saline','Balanced'), labelcol=c('red','blue')))
mtext('Total isotonic crystalloid through day 3', side=1, line=2.5,cex=1)
mtext('Incidence of MAKE30', side=2, line=2.5,cex=1)
text(10000,0.9,paste0('P-value for interaction = ',sprintf("%4.3f",anova(mq)[2,3])), adj=1)
dev.off()


# Interaction between study groups and volume crystalloid received with regard to chloride concentration
# supplemental figure
pdf('fig7.pdf')
par(mar=c(4,4,1,1))
with(fulldata, LoessWithCI(isotonic.crystalloid.cum30,highestdaily.cl.max,group=group,
                           col=c('red','blue'), side=c(3,1), xlim=c(0,10000), ylim=c(105,120), xlab='',
                           ylab='', hists=TRUE, cex.axis=1.5, textsize=1.5,
                           curvelabprint=TRUE, labellab=c('Saline','Balanced'),
                           labelcol=c('red','blue')))
mtext('Total isotonic crystalloid through day 30 (mL)', side=1, line=2.5,cex=1)
mtext('Highest chloride (mmol/L)', side=2, line=2.5,cex=1)
text(10000,119,paste0('P-value for interaction = ',sprintf("%4.3f",
            anova(lrm(highestdaily.cl.max ~ group * rcs(isotonic.crystalloid.cum30,3),
                      data = fulldata, x = TRUE, y = TRUE))[2,3])), adj=1)
dev.off()

# Compliance with assigned fluid by volume of fluid received - supplemental figure
pdf('fig8.pdf')
par(mfrow=c(1,1),mar=c(4,4,1,1))
with(fulldata, LoessWithCI(isotonic.crystalloid.cum30,saline.prop,group=group, col=c('red','blue'),
                           side=c(3,1), xlim=c(0,10000), ylim=c(0,1), xlab='', ylab='', hists=TRUE,
                           cex.axis=1.5, textsize=1.5, curvelabprint=TRUE,
                           labellab=c('Saline','Balanced'), labelcol=c('red','blue')))
mtext('Total isotonic crystalloid through day 30 (mL)', side=1, line=2.5,cex=1)
mtext('Proportion of saline among total isotonic crystalloid received', side=2, line=2.5,cex=1)
dev.off()
```

```
# Interaction between study groups and volume crystalloid received with regard to bicarbonate
# concentration - supplemental figure
pdf('fig9.pdf')
par(mar=c(4,4,1,1))
with(fulldata, LoessWithCI(isotonic.crystalloid.cum30,lowestdaily.co2.min,group=group,
                           col=c('red','blue'), side=c(3,1), xlim=c(0,10000), ylim=c(12,22), xlab='',
                           ylab='', hists=TRUE, cex.axis=1.5, textsize=1.5, curvelabprint=TRUE,
                           labellab=c('Saline','Balanced'), labelcol=c('red','blue')))
mtext('Total isotonic crystalloid through day 30 (mL)', side=1, line=2.5,cex=1)
mtext('Lowest bicarbonate concentration', side=2, line=2.5,cex=1)
p<-anova(lrm(lowestdaily.co2.min ~ group * rcs(isotonic.crystalloid.cum30,3),
             data = fulldata, x = TRUE, y = TRUE))[2,3]
text(10000,21,ifelse(p<0.001, 'P-value for interaction <.001',
                     paste0('P-value for interaction = ',sprintf("%4.3f",p))), adj=1)
dev.off()


# Interaction between study groups and volume crystalloid received with regard to highest daily
# creatinine - supplemental figure
pdf('fig10.pdf')
par(mar=c(4,4,1,1))
with(fulldata, LoessWithCI(isotonic.crystalloid.cum30,highestdailycreat.max,group=group,
                           col=c('red','blue'), side=c(3,1), xlim=c(0,10000), ylim=c(1,5), xlab='',
                           ylab='', hists=TRUE, cex.axis=1.5, textsize=1.5, curvelabprint=TRUE,
                           labellab=c('Saline','Balanced'), labelcol=c('red','blue')))
mtext('Total isotonic crystalloid through day 30 (mL)', side=1, line=2.5,cex=1)
mtext('Highest creatinine concentration (mg/dL)', side=2, line=2.5,cex=1)
p<-anova(lrm(highestdailycreat.max ~ group * rcs(isotonic.crystalloid.cum30,3),
             data = fulldata, x = TRUE, y = TRUE))[2,3]
text(10000,4.8,ifelse(p<0.001, 'P-value for interaction <.001',
                      paste0('P-value for interaction = ',sprintf("%4.3f",p))), adj=1)
dev.off()



# Interaction between study groups and volume crystalloid received with regard to AKI -
# supplemental figure
pdf('fig11.pdf')
par(mar=c(4,4,1,1))
with(fulldata, LoessWithCI(isotonic.crystalloid.cum30,incidentAKI,group=group, col=c('red','blue'),
                           side=c(3,1), xlim=c(0,10000), ylim=c(0,1), xlab='', ylab='', hists=TRUE,
                           cex.axis=1.5, textsize=1.5, curvelabprint=TRUE,
                           labellab=c('Saline','Balanced'), labelcol=c('red','blue')))
p<-anova(lrm(incidentAKI ~ group * rcs(isotonic.crystalloid.cum30,3),
             data = fulldata, x = TRUE, y = TRUE))[2,3]
mtext('Total isotonic crystalloid through day 30 (mL)', side=1, line=2.5,cex=1)
mtext('Rate of incident AKI', side=2, line=2.5,cex=1)
text(10000,0.95,ifelse(p<0.001, 'P-value for interaction <.001',
                       paste0('P-value for interaction = ',sprintf("%4.3f",p))), adj=1)
dev.off()



# Interaction between study groups and volume crystalloid received with regard to new RRT -
# supplemental figure
pdf('fig12.pdf')
par(mar=c(4,4,1,1))
with(fulldata, LoessWithCI(isotonic.crystalloid.cum30,new.rrt.verified,group=group, col=c('red','blue'),
                           side=c(3,1), xlim=c(0,10000), ylim=c(0,0.3), xlab='', ylab='', hists=TRUE,
```

```
                          cex.axis=1.5, textsize=1.5, curvelabprint=TRUE,
                          labellab=c('Saline','Balanced'), labelcol=c('red','blue')))
mtext('Total isotonic crystalloid through day 30 (mL)', side=1, line=2.5,cex=1)
mtext('Receipt of RRT', side=2, line=2.5,cex=1)
p<-anova(lrm(new.rrt.verified ~ group * rcs(isotonic.crystalloid.cum30,3),
             data = fulldata, x = TRUE, y = TRUE))[2,3]
text(10000,0.28,ifelse(p<0.001, 'P-value for interaction <.001',
                       paste0('P-value for interaction = ',sprintf("%4.3f",p))), adj=1)
dev.off()



# Interaction between study groups and volume crystalloid received with regard to death
# Supplemental figure
pdf('fig13.pdf')
par(mar=c(4,4,1,1))
with(fulldata, LoessWithCI(isotonic.crystalloid.cum30,make30.death,group=group, col=c('red','blue'),
                          side=c(3,1), xlim=c(0,10000), ylim=c(0,0.45), xlab='', ylab='', hists=TRUE,
                          cex.axis=1.5, textsize=1.5, curvelabprint=TRUE,
                          labellab=c('Saline','Balanced'), labelcol=c('red','blue')))
p<-anova(lrm(make30.death ~ group * rcs(isotonic.crystalloid.cum30,3),
             data = fulldata, x = TRUE, y = TRUE))[2,3]
mtext('Total isotonic crystalloid through day 30 (mL)', side=1, line=2.5,cex=1)
mtext('Mortality', side=2, line=2.5,cex=1)
text(10000,0.4,ifelse(p<0.001, 'P-value for interaction <.001',paste0('P-value for interaction = ',
                                                                    sprintf("%4.3f",p))), adj=1)
dev.off()





## Primary Analysis
m1 <- glmer(make30.final ~ group + patient.age + patient.gender + patient.race.v2 +
              source.of.admission.v2 + ventilator.flag + inotropic.flag + Sepsis + TBI + (1 | unit),
            data = fulldata, family = binomial(link=logit))
# compute confidence interval
cc <- confint(m1,parm="beta_")
ctab <- cbind(est=fixef(m1),cc)
latex(ctab, file='', where='!h')



## Sensitivity Analysis - Missing Creatinine Measures
labs <- csv.get('LabsByDay.csv', lowernames=TRUE, header = TRUE, sep = ",", na.strings = c("","NA","NULL"))
labs <- subset(labs, encounter.number %in% fulldata$encounternumber)
names(labs)[2] <- 'encounternumber'

creat.all <- labs[,c('encounternumber', paste0('firstdailycreat.',0:30), paste0('lowestdailycreat.',0:30),
                     paste0('highestdailycreat.',0:30))]

creat.all$max.creat <- ifelse(apply(creat.all[,2:94], 1, function(x)sum(is.na(x)))==93, NA,
                              apply(creat.all[,2:94], 1, function(x)max(x, na.rm=TRUE)))
creat.all$min.creat <- ifelse(apply(creat.all[,2:94], 1, function(x)sum(is.na(x)))==93, NA,
                              apply(creat.all[,2:94], 1, function(x)min(x, na.rm=TRUE)))

creat.first=reshape(labs[,c('encounternumber',paste0('firstdailycreat.',0:30))],
                    varying=list(2:32), idvar='encounternumber', times=0:30, direction='long')
creat.first <- subset(creat.first, !is.na(firstdailycreat.0))
```

```
creat.first <- creat.first[order(creat.first$encounter, creat.first$time),]
ff = tapply(creat.first[["firstdailycreat.0"]], creat.first$encounternumber, head, n=1)
creat.first[["first.creat"]] = ff[as.character(creat.first$encounternumber)]
creat.first <- unique(creat.first[,c('encounternumber','first.creat')])

fulldata <- merge(fulldata, creat.all[,c('encounternumber','min.creat','max.creat')],
                  by='encounternumber', all.x=TRUE)
fulldata <- merge(fulldata, creat.first, by='encounternumber', all.x=TRUE)
fulldata <- upData(fulldata,
                   make30.firstcreat = ifelse(make30.death == 1 |
                                              new.rrt.verified ==1 |
                                              make30.finalstudycreat/first.creat>=2, 1, 0),
                   make30.mincreat = ifelse(make30.death == 1 |
                                            new.rrt.verified ==1 |
                                            make30.finalstudycreat/min.creat>=2, 1, 0),
                   make30.maxcreat = ifelse(make30.death == 1 |
                                            new.rrt.verified ==1 |
                                            make30.finalstudycreat/max.creat>=2, 1, 0),
                   make30.creat1.5=ifelse(make30.death == 1 |
                                          new.rrt.verified ==1 |
                                          make30.finalstudycreat/make30.baselinecr>=1.5, 1, 0))


############### Imputation

temp <- fulldata
temp$basecreat1 <- ifelse(temp$make30.baselinecrsource=='Calculated', NA, temp$make30.baselinecr)
temp$basecreat2 <- ifelse(temp$make30.baselinecrsource %in%
                          c('Calculated','LowestCreatDurEncPriorTo_ICU'), NA, temp$make30.baselinecr)
temp$race2 <- temp$patient.race
levels(temp$race2) <- list('WHITE'='WHITE', 'BLACK'='BLACK',
                           'OTHER'=c('ALASKAN/INDIAN','ASIAN', 'DECLINED', 'PACIFIC ISLAND','UNKNOWN/TBD'))

creat.impute <- aregImpute( ~ patient.age + patient.gender + race2 + group + source.of.admission.v2 +
                              Sepsis + TBI + ventilator.flag + inotropic.flag + hemodialysis.pre.icu +
                              isotonic.crystalloid.cum30 + uhc.expected.mort + make30.death +
                              new.rrt.verified + min.creat + max.creat + make30.finalstudycreat +
                              basecreat1, x=TRUE, data=temp, burnin=2, n.impute=5)
creat.imputed <- as.data.frame(creat.impute$x)

creat.impute2 <- aregImpute( ~ patient.age + patient.gender + race2 + group + source.of.admission.v2 +
                               Sepsis + TBI + ventilator.flag + inotropic.flag + hemodialysis.pre.icu +
                               isotonic.crystalloid.cum30 + uhc.expected.mort + make30.death +
                               new.rrt.verified + min.creat + max.creat + make30.finalstudycreat +
                               basecreat2, x=TRUE, data=temp, burnin=2, n.impute=5)
creat.imputed2 <- as.data.frame(creat.impute2$x)

fulldata=cbind(fulldata,creat.imputed[,18],creat.imputed2[,18])

fulldata <- upData(fulldata,
                   make30.imp.cal = ifelse(make30.death == 1 |
                                           new.rrt.verified ==1 |
                                           make30.finalstudycreat/creat.imputed[, 17]>=2, 1, 0),
                   make30.imp2 = ifelse(make30.death == 1 | new.rrt.verified ==1 |
                                        make30.finalstudycreat/creat.imputed2[, 17]>=2, 1, 0))


tt <- datadist(fulldata[,c('make30.final','group')]); options(datadist = 'tt')
```

```r
ImpVar <- c('make30.final','make30.imp.cal','make30.imp2','make30.firstcreat','make30.maxcreat',
            'make30.mincreat','make30.creat1.5')

SenAnalysis <- NA
for (i in 1:length(ImpVar)){

   mmod <- glmer(as.formula(paste(ImpVar[i], "group + patient.age + patient.gender +
                                  patient.race.v2 + source.of.admission.v2 + ventilator.flag +
                                  inotropic.flag + Sepsis + TBI + (1 | unit)", sep=" ~ ")),
                data = fulldata, family = binomial)

    cc <- confint(mmod,parm="beta_")
    SenAnalysis <- rbind(SenAnalysis,c(exp(cbind(est=fixef(mmod),cc)[2,]),
                                        summary(mmod)$coefficients[2,4]))
}

mtemp <- glmer(make30.final ~ group + patient.age + patient.gender +
                                  patient.race.v2 + source.of.admission.v2 + ventilator.flag +
                                  inotropic.flag + Sepsis + TBI + (1 | unit),
                                  data = subset(fulldata,!make30.baselinecrsource=='Calculated'),
                                  family = binomial)
cc <- confint(mtemp,parm="beta_")
SenAnalysis <- rbind(SenAnalysis[-1,],
                 c(exp(cbind(est=fixef(mtemp),cc)[2,]),
                                        summary(mtemp)$coefficients[2,4]))

rownames(SenAnalysis) <- c('ITT','Single imputation on missing baseline creatinine',
                           'Single imputation treating LowestCreatDurEncPriorToICU missing',
                           'Use first creatinine','Use highest creatinine','Use lowest creatinine',
                           'Use ratio 1.5 instead of 2','Complete cases')
kable(outtemp, digits=2, caption='Sensitivity Analysis Using Different Renal Dysfunction Calculation',
      align=rep('c',4), escape=FALSE, format='latex')




# All other sensitivity analysis
# 1. Modified intention-to-treat population: patients who received at least 500 mL of isotonic
#    crystalloid in the 72 hours after enrollment
AnalysisSet <- subset(fulldata, mod.itt=='Yes')

# 2. Washout: excluding pateints admitted within the 7 days prior to a cross-over in ICU fluid group
#             assignment
AnalysisSet <- subset(fulldata, crossover7=='No')

# 3. Per protocol: excluding patients who remained in the ICU through a cross-over or were transferred
#                  between study ICUs
fulldata <- subset(fulldata, crossover=='No')

# 4. Repeat same analysis including only the first admission per patient
```
